# Engineering a cleaved, prefusion-stabilized influenza B virus hemagglutinin by identification and locking of all six pH switches

**DOI:** 10.1093/pnasnexus/pgae462

**Published:** 2024-10-11

**Authors:** Jarek Juraszek, Fin J Milder, Xiaodi Yu, Sven Blokland, Daan van Overveld, Pravien Abeywickrema, Sem Tamara, Sujata Sharma, Lucy Rutten, Mark J G Bakkers, Johannes P M Langedijk

**Affiliations:** Janssen Vaccines & Prevention BV, 2333 CN Leiden, The Netherlands; Janssen Vaccines & Prevention BV, 2333 CN Leiden, The Netherlands; Structural and Protein Science, Janssen Research and Development, Spring House, PA 19044, USA; Janssen Vaccines & Prevention BV, 2333 CN Leiden, The Netherlands; Janssen Vaccines & Prevention BV, 2333 CN Leiden, The Netherlands; Structural and Protein Science, Janssen Research and Development, Spring House, PA 19044, USA; Janssen Vaccines & Prevention BV, 2333 CN Leiden, The Netherlands; Structural and Protein Science, Janssen Research and Development, Spring House, PA 19044, USA; Janssen Vaccines & Prevention BV, 2333 CN Leiden, The Netherlands; Janssen Vaccines & Prevention BV, 2333 CN Leiden, The Netherlands; Janssen Vaccines & Prevention BV, 2333 CN Leiden, The Netherlands

## Abstract

Vaccine components based on viral fusion proteins require high stability of the native prefusion conformation for optimal potency and manufacturability. In the case of influenza B virus hemagglutinin (HA), the stem's conformation relies on efficient cleavage. In this study, we identified six pH-sensitive regions distributed across the entire ectodomain where protonated histidines assume either a repulsive or an attractive role. Substitutions in these areas enhanced the protein's expression, quality, and stability in its prefusion trimeric state. Importantly, this stabilization enabled the production of a cleavable HA0, which is further processed into HA1 and HA2 by furin during exocytic pathway passage, thereby facilitating correct folding, increased stability, and screening for additional stabilizing substitutions in the core of the metastable fusion domain. Cryo-EM analysis at neutral and low pH revealed a previously unnoticed pH switch involving the C-terminal residues of the natively cleaved HA1. This switch keeps the fusion peptide in a clamped state at neutral pH, averting premature conformational shift. Our findings shed light on new strategies for possible improvements of recombinant or genetic-based influenza B vaccines.

**Significance Statement**

Influenza B virus hemagglutinin (HA) is a pH-sensitive metastable class I viral fusion protein that undergoes dramatic conformational changes between pre- and postfusion states. While a critical component of influenza vaccines, real-world data and clinical vaccine studies indicate suboptimal performance for the B component, prompting the development of next-generation designs. Recognizing the significance of stabilizing prefusion conformations for effective immune responses, we employed a structure-based approach to improve influenza virus B HA. We identified and locked six pH-sensitive switches distributed across the protein. Introducing stabilizing substitutions in and around these switches facilitated the production of high-quality native prefusion protein at high levels. This innovative strategy ensures the consistent production of top-tier HA B components for next-generation influenza vaccines.

## Introduction

Influenza viruses significantly contribute to the morbidity and mortality associated with viral respiratory diseases (1). The fusion and receptor-binding protein hemagglutinin (HA) is a critical component of commercial vaccines, emphasizing the importance of a comprehensive understanding of its stability (2, 3). Viral fusion proteins mediate fusion of viral and host cell membranes, thereby facilitating viral entry and replication within the host cell. The transformation of the fusion proteins from the prefusion to the postfusion state is a critical step in viral entry, and this transition irreversibly alters the protein's antigenic features. The triggers for this conformational change can be diverse and may include proteolytic cleavage, exposure to low pH, or interaction with target cell receptors. Inherent to their role in this process, fusion proteins must adopt a metastable, high-energy prefusion conformation. This intrinsic metastability can lead to low expression levels, impediments in the formation of correctly folded prefusion trimers, and reduce potency and shelf life of protein-based vaccines.

**Fig. 1.**
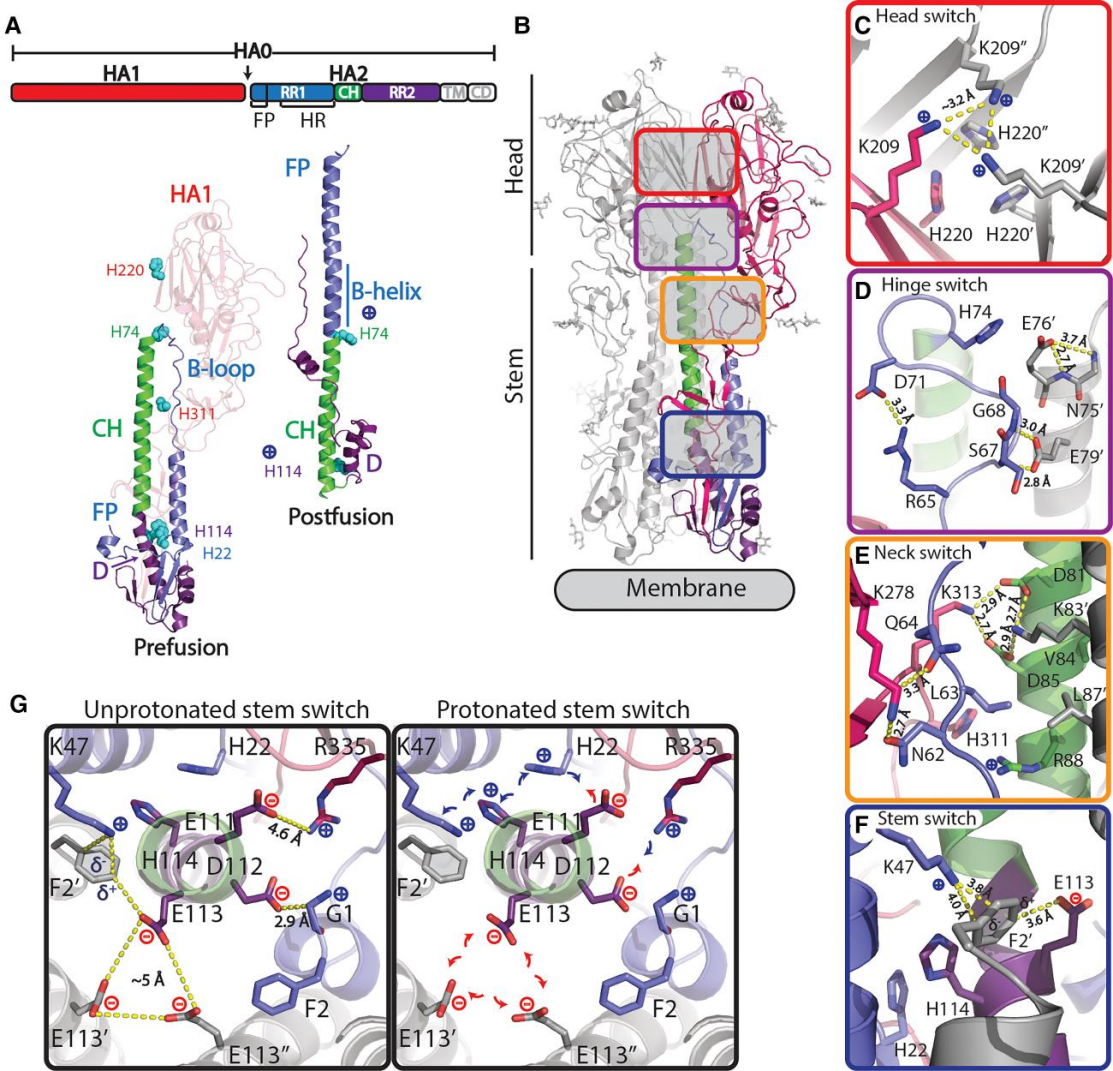
Influenza B virus HA protein structure. A) Schematic representation of HA with indicated RR1, including FP and HR, RR2, CH, transmembrane domain, and cytoplasmic domain. Structures of influenza B virus monomer in prefusion (26) and postfusion (27) conformation colored according to upper schematic description. B-loop and B-helix are indicated and histidines (H) in regions of instability. B) Structure of trimeric prefusion HA with one protomer colored according to A and location of regions of instability with zoomed-in colored panels to the right of the C) head switch (top view) and D) hinge, E) neck, and F) stem switch (side view). G) Top view of stem switch at the plane of the helical kink of helix C and D showing the repulsive cluster with E113 and the two pH-sensitive switches around H22 and H114 in an unprotonated situation (left panel) and possible impact after protonation (right panel). Colored according to A.

Stabilization of the prefusion conformation has emerged as a key success factor for the induction of efficacious immune responses by several vaccines. As a result, stabilization has become a significant focus of the emerging field of structural vaccinology (4–12). When it egresses from the cell, influenza HA0 is cleaved into the head domain (HA1) and the membrane anchored fusion domain (HA2) to become a fusion competent HA1/2 heterotrimer (13–16). HA2 consists of the N-terminal refolding region 1 (RR1) that contains the fusion peptide (FP) and a heptad repeat (HR) motif (residues HA2 1 to 75, using the influenza B numbering), the central helix (CH, residues HA2 76 to 105), and the C-terminal refolding region 2 (RR2, residues HA2 106 to 184; Fig. 1A). The HR motif in RR1 corresponds to helix A—loop—helix B. Following sialic acid binding and uptake into the endosome, the low pH triggers the release of the FP from the pocket located at the trimer interface, which is then inserted into the target membrane. In the second stage, the stem bends at a kink located at the end of the CH, resulting in reorganization of RR2 which subsequently docks onto the elongated RR1 coiled-coil, bringing the two membranes together (Fig. 1A) (17–25).

While the refolding of influenza HA is principally triggered by low pH, the protein is also metastable under physiological conditions (23, 28).

In contrast to influenza A, an additional challenge for influenza B virus HA is that the correct native conformation relies on HA1/HA2 processing, which allows for native folding of the stem without splaying at the membrane-proximal base (Fig. S1). Notably, the FP only docks in the trimer interface and enables the folding of a native compact stem structure postcleavage. Although a critical ingredient for a broad protection against influenza infections (1, 29), in many commercial vaccines, the influenza B virus HA component is suboptimal because full cleavage is not reached (30). To produce cleaved HA without requiring the addition of exogenous trypsin, a furin cleavage site can be introduced that enables efficient intracellular cleavage at low pH during passage through the exocytic pathway by subtilisin-like proteases. To abrogate pH-induced conformational changes that can occur after proteolytic processing in the acidic exosomal pathway, HA needs to be stabilized in the prefusion conformation.

Previously, stabilization of influenza A group 1 and 2 HAs was achieved by introducing mutations in two pH-sensitive switch regions involved in the early refolding process (28). However, no stabilization approach is described for influenza B virus HA, which shares only about 25% sequence identity with influenza A. Although the pH-sensitive switches found in influenza A are not conserved in influenza B, and although the refolding mechanism of influenza B virus HA is hardly studied, several regions have been identified that feature conserved ionizable residues, potentially causing instability and triggering conformational change upon exposure to low pH (13). Here, we characterized the low stability of wild-type (WT) influenza B virus HAs and achieved stabilization through substitutions in five regions of instability that are involved in the protein refolding. Cryogenic electron microscopy (cryo-EM) of the stabilized and cleaved HA explained the impact of the stabilizing substitutions and additionally uncovers a sixth region of instability—a previously overlooked pH switch that clamps the FP at neutral pH. This stabilization approach proved effective for all tested HAs from different influenza B strains, especially when combined with an occasional back mutation of rare amino acids to consensus, a process we termed “repair,” and which we previously applied to human immunodeficiency virus-1 Env and influenza A HA (10, 28).

## Results

### Stabilization of HA by substitutions in regions of instability

To obtain a cleaved stabilized influenza B virus HA, we conducted an in-depth analysis of the protein structure in search for regions of instability. Recognizing that the refolding of influenza HA is triggered by a drop in pH, we focused on conserved, ionizable, and at least partially buried residues that might be involved in initiating the refolding process.

Similar to influenza type A, influenza B virus HAs feature clusters of histidines and charged residues, forming pH-sensitive switches, or simply pH switches. These clusters are not conserved between influenza A and B, and are located in multiple regions—head (HA1-His220), hinge (HA2-His74), neck (HA1-His311), and stem (HA2-His22 + His114; Fig. 1B–G). In the hinge switch (Fig. 1D), HA2 Glu76 caps the CH, likely preventing conformational change by hindering B-loop extension. Upon protonation, HA2-His74 can attract Glu76, weakening the helical N-cap and easing B-loop refolding. Nearby, in the neck, an ionizable residue cluster potentially destabilizes the B-loop (Fig. 1E). The stem pH switch at the kink between helix C and D (Fig. 1F and G) is part of an intricate network of stabilizing interactions involving multiple regions in HA that are involved in refolding, including the FP. Cleavage of HA0 facilitates the FP's interaction with the trimer interface and results in a tightly packed trimeric stem. HA2 Phe2 is a key in anchoring the FP and balancing charges at the interface, particularly between HA2 Glu113 and Lys47. Notably, the anion—aromatic interaction of Glu113 with Phe2 (31), allows for the formation of the metastable cluster of Glu113 in the center of the stem. Stabilization of the Phe2, achieved primarily through the electrostatic rather than hydrophobic or stacking interactions, enables FP release, as protonation of the adjacent His114 can weaken these electrostatic interactions. FP release could also be influenced by the charge interaction network involving HA2-His22, Glu111, and HA1 Arg 335, affecting FP—stem interactions and potentially leading to stem splaying, known as an early refolding step (19, 22, 32, 33).

Based on this detailed analysis of the regions of instability, we designed and tested point mutations for their capacity to stabilize influenza B virus HA. Substitutions were identified through both visual inspection and guided by the utilization of Rosetta's mutant design, following the coupled-moves protocol (CMP) (34) as described (35), which accommodates backbone flexibility. We employed three structures for the design of stabilizing substitutions: B/Yamanashi/166/1998 (PDB ID 4M44, B/Yamagata/16/88-like lineage), B/Lee/40 (PDB ID 4NRJ), and B/Brisbane/60/2008 (PDB ID 6FYW, B/Victoria/2/87-like lineage) (26, 36, 37). We focused on mutations situated in the regions of interest that inactivate the potential pH switches. These were chosen based on a positive CMP score for at least two structures and a nonnegative score across all the analyzed structures (Supplementary data file CMP score). To stabilize the head, we introduced several substitutions at and around residues K209 and H220 of HA1. These changes aimed to mitigate the possible charge repulsion caused by a protonated Lys209 and His220. For the hinge and neck switch, we introduced substitutions in the vicinity of H74 of HA2 and H311 of HA2. These substitutions are designed to lock the position of the B-loop in RR1, and to impede the hinge movement and the subsequent formation of the extended helix. The repulsive Glu113 cluster and the complex stem pH switches were stabilized through various substitutions in HA2 at position 113, and substitution of the histidines at positions 22 and 114. Based on the structural consideration and the CMP scores for the 3 structures, 83 single-point mutations were selected.

**Fig. 2.**
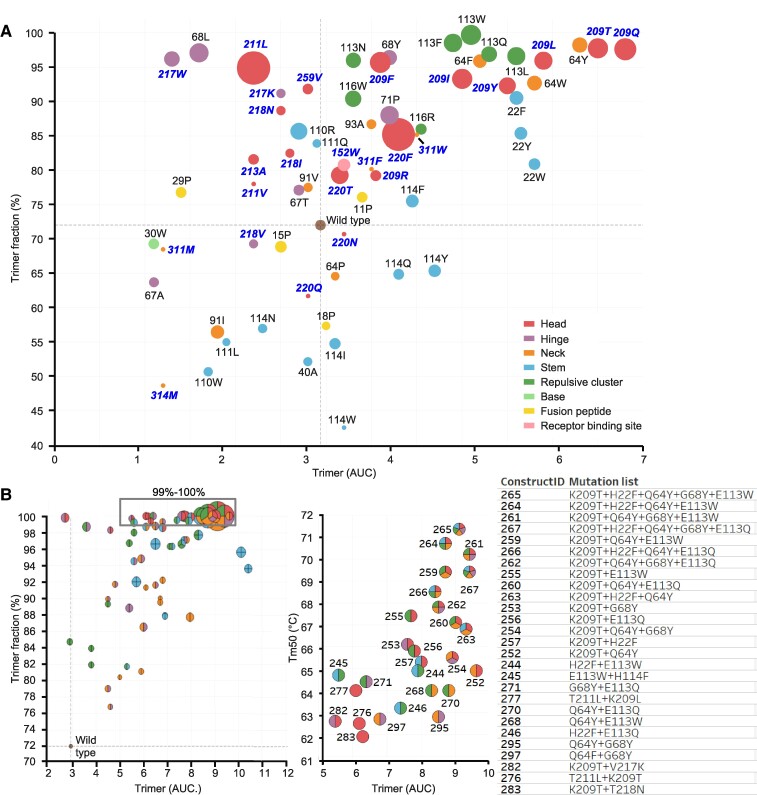
Characterization of HA variants containing stabilizing substitutions. HA trimer expression using analytical SEC and thermal stability using DSF of cell culture supernatant of Expi293F cells expressing influenza B virus HA (Fig. S2). A) A plot of trimer expression level against trimer:monomer ratio for each mutant is shown. The radius of markers is relative to the melting temperatures (Tm_50_) as measured by DSF. WT is indicated in brown at the cross hair. Markers are colored by different regions, including the regions of instability. Labels show residue number for HA1 (bold italic in blue) and HA2 (black). B) Plot of trimer expression level against trimer:monomer ratio for combinations of stabilizing substitutions with marker radius relative to Tm_50_. Markers are shown as pie charts with the colors according to the region of the stabilizing substitution as in (A) (left panel). Fully trimeric variants are boxed in the left panel and represented in the right panel plot showing trimer expression level against Tm_50_. Markers are colored pie charts with color shades corresponding to the region of the stabilizing mutations.

The impact of the single substitutions on the stability of soluble HA was tested using influenza B/Iowa/06/2017. Supernatants of cells transfected with plasmids encoding HA variants were assessed for trimer content using size-exclusion chromatography (SEC) and thermostability was measured using differential scanning fluorimetry (DSF). In all five regions of instability, substitutions were identified that increased trimer content and thermal stability compared with WT HA (Figs. 2A and S2, Table S1). Notably, substitutions in the head switch—and especially substitutions of K209—demonstrated the most significant increases in trimer expression, trimer-to-monomer ratio, and thermal stability. All substitutions targeting the potentially repulsive cluster formed by the three E113 residues in the trimer interface yielded an increase in stability of the quaternary structure. In the neck switch, stabilization was accomplished through adjacent substitutions like Q64Y, rather than directly targeting H311. The D71P substitution in the B-loop also had a stabilizing effect, echoing the effect of similar stabilizing proline substitutions observed in class I fusion protein hinge loops (4–10). Additionally, we found that various combinations of substitutions selected from the different regions led to complete trimer formation and further boosted expression levels (Fig. 2B, left panel). For the boxed selection of variants that formed full trimers in Fig. 2A, the expression levels were plotted against the melting temperatures (Figs. 2B, right panel, and S3). A cumulative effect was observed when substitutions in all five unstable regions were combined, achieving the highest expression levels and melting temperatures reaching Tm_50_ values of over 70 °C (Fig. 2B).

### Stabilization of cleaved HA of multiple influenza B strains

In contrast to influenza A, in which the uncleaved and cleaved HA have a very similar structure, the uncleaved influenza B virus HA is splayed at the base and has a relocated FP and is antigenically very different from the cleaved influenza B virus HA which has a compact and closed stem (Figs. 1 and S1). As a result, any stabilizing substitution in the stem region would be more accurately assessed in a cleaved HA. However, most cells, including HEK293F cells, do not support cleavage at the typical monobasic cleavage site of influenza B virus HA.

**Fig. 3.**
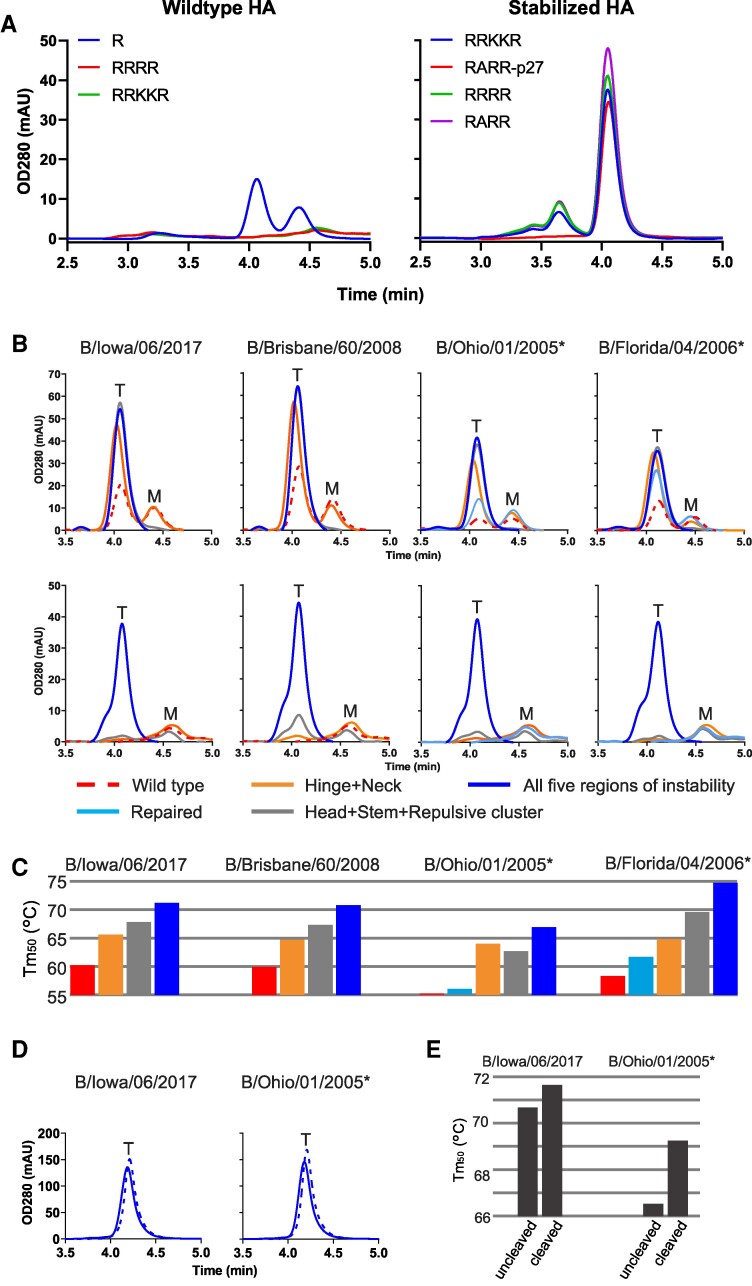
Stability of cleaved and uncleaved HAs. A) Impact of introduction of furin cleavage sites to analytical SEC profiles of culture supernatants of Expi293F cells expressing influenza B virus HA variants based on WT influenza B virus HA with monobasic or polybasic cleavage sites (left panel) or HA stabilized by substitutions in five regions of instability with different furin site designs (right panels). B) Impact of different levels of stabilization on trimer formation in uncleaved (upper panels) and cleaved (lower panels) HA variants for four different influenza B strains. Analytical SEC profiles of culture supernatant of Expi293F cells expressing selected influenza B WT (red broken line) repaired (light blue line in right upper panels), stabilization of neck and hinge switch (HA1 H311W and HA2 Q64W), stabilization of repulsive cluster, head, and stem switch (HA2 E113W, HA1 K209T, and HA2 H22F) or stabilization in five regions of instability (HA1 K209T, HA2 H22F, Q64Y, G68Y, and E113W). Upper panels contain a monobasic cleavage site which is not cleaved. Bottom panels are cleaved variants with an engineered RSV p27 domain which is cleaved out by furin at low pH. Peaks representing monomeric and trimeric species are indicated by an “M” and a “T,” respectively. C) Temperature stability as determined by DSF for WT and stabilized uncleaved HA variants. Colors as in B. D) Analytical SEC profiles of purified uncleaved (solid line) and cleaved (broken line) stabilized B/Iowa/06/2017 and B/Ohio/01/2005 HA. E) Temperature stability as determined by DSF of purified uncleaved and cleaved stabilized HAs. Stabilized variants including repair mutations are indicated by the asterisk symbol. All strains in the panels are from Victoria lineage except for B/Florida/04/2006 which is from Yamagata lineage.

To facilitate screening of trimer yield, thermal stability and pH stability in natively cleaved HA, a furin site was introduced to enable cleavage of HA0 during its passage through the exocytic pathway in Expi293F cells. We investigated several cleavage sites: a polybasic cleavage site, a signature polybasic cleavage site as observed in highly pathogenic avian influenza A HA, and the p27 domain of the respiratory syncytial virus (RSV) fusion protein, which is flanked by two furin sites and efficiently processed upon exosomal transport (38, 39). No HA could be detected when furin cleavage sites were introduced into WT unstabilized HA since upon cleavage, HA will be triggered and undergo the conformational change to the postfusion state (Fig. 3A, left panel). However, pH instability was completely mitigated when substitutions were introduced in the five regions of instability. The introduction of furin cleavage sites in stabilized HA led to high expression of trimeric cleaved HA (Fig. 3A, right panel). HA with the p27 peptide insertion was most effectively cleaved, resulting in a trimer peak without the presence of any additional higher molecular weight species. To explore the general applicability of these stabilizing mutations, we assessed their effect in several HA variants from the B/Victoria and B/Yamagata lineage with sequence identity of 93% or higher. HAs were either partially stabilized in the neck region by HA1 H311W and HA2 Q64W, partially stabilized in the head, repulsive cluster, and the stem region (HA1 K209T, HA2 E113W, and HA2 H22F), or fully stabilized across all five regions of instability (HA1 K209T, HA2 H22F, Q64Y, G68Y, and E113W). We created these variants for four different B strains in both noncleavable and furin cleavable forms, incorporating a p27 domain that gets cleaved in the low pH compartment (Fig. 3B and C). In order to obtain a native HA1 C-terminus, we did not introduce the additional ARR sequence N-terminal to p27 and we co-transfected 10% furin in order to obtain efficient cleavage at the monobasic cleavage site. It is worth noting that some sequences obtained from the Global Initiative on Sharing all Influenza Data initiative, or the influenza virus database contain rare, strain-specific residues. These may arise from, e.g. egg adaptations (3) or sequencing errors and may require optimization or “repair” to ensure correct and efficient folding (3, 10, 28). Specifically, the HAs of B/Ohio/01/2005 and B/Florida/04/2006 showed elevated trimer expression when two exceptionally rare residues were substituted with the consensus influenza B virus HA amino acids (Table S2 and upper right panels in Fig. 3B). Uncleaved HAs stabilized in the neck region exhibited increased trimer expression and thermostability (Fig. 3B and C). Uncleaved variants stabilized in either three or all five regions of instability showed high trimer expression without any monomeric forms and increased thermostability. For cleavable HA, only the variant stabilized in all five regions withstood the low-pH conditions of the exocytotic pathway and yielded high trimeric HA expression across all four influenza B strains (Fig. 3B, lower panels). To further scrutinize the role of the FP's native location in the interface of the cleaved HA trimer, we purified both uncleaved and cleaved influenza B virus HA with all five stabilizing substitutions (Fig. 3D). We then evaluated the proteins’ thermostability using DSF (Fig. 3E). Remarkably, despite the five stabilizing substitutions, the added stability conferred by the FP interaction remained measurable in the cleaved HA variants. This corroborates the structural importance of the FP and the correct folding of a natively closed stem region.

**Fig. 4.**
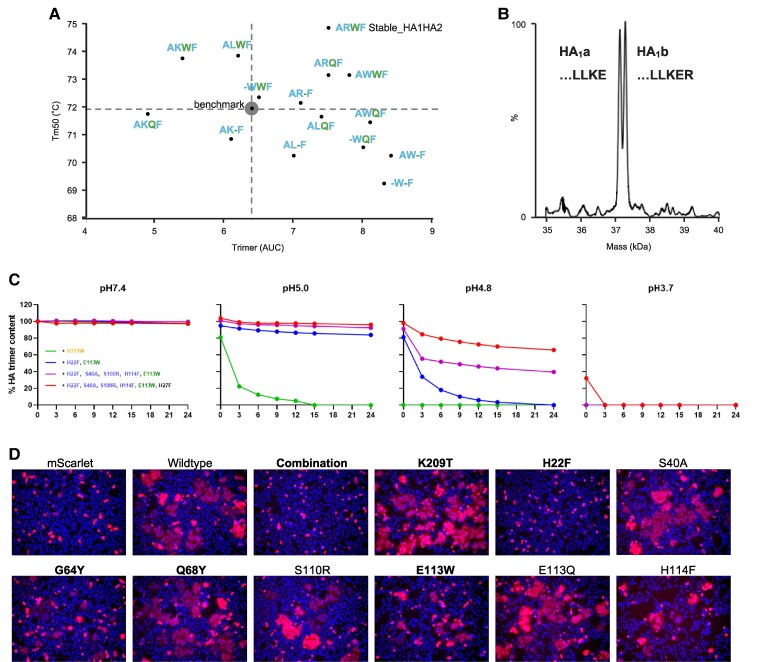
The characterization of additional HA substitutions in the stem switch and repulsive cluster region. Analytical SEC and DSF of cell culture supernatant of Expi293F cells expressing variants of stabilized cleaved B/Iowa/06/2017 HA. A) All variants contained identical stabilizing substitutions (HA1 K209T, HA2 Q64Y, G68Y, and H22F) and variations at several positions in the stem switch (S40A, S110R, and H114F) and repulsive cluster (E113W/Q). Plot showing cleaved influenza B virus HA trimer expression level against temperature stability (Tm_50_), compared with benchmark stabilized HA indicated with dashed lines and gray sphere (HA1 K209T, HA2 E113W, Q64Y, G68Y, and H22F). The labels indicate substitutions at positions HA2 40, 110, 113, and 114 using the color coding as in Fig. 2. B) Intact mass LC–MS analysis of HA1 chain from purified Stable_HA1HA2 labeled ARWF, as described in Fig. S5. HA1 is present as equally abundant forms HA1a and HA1b with and without C-terminal arginine. C-terminal sequence is indicated in the plot. C). pH stability in different buffers during 24 h of HA variants stabilized in head, hinge, and neck (green line), stabilized in five regions of instability (blue line), as previous with three additional stabilizing substitutions in the stem (purple line) and as previous including H27F substitution in the sixth region of instability (red line). All constructs include (HA1 K209T, HA2 G68Y, and Q64Y), Additional substitutions are indicated in the left panel for each construct with color coding as in Fig. 2. HA trimer content is indicated as a percentage of the starting material as measured by analytical SEC. For pH 4.8, the raw data are shown in Fig. S9. D) HA fusogenicity as measured in a cell–cell fusion assay in HEK293 cells by co-transfection of plasmids encoding WT or stabilized HA, human TMPRSS2, and mScarlet. Fusion was triggered 24 h posttransfection by a 10-min exposure to pH 5.0 medium, followed by a 1-h recovery at pH 7.4. Shown are overlays of red and blue (Hoechst) channels. The “combination” design has all stabilizing substitutions indicated in bold.

The instability of cleaved HA at low pH, created an opportunity to measure additional stabilizing substitutions that did not have an additive stabilizing effect on uncleaved HA. Therefore, an additional set of stabilizing substitutions in the stem region at positions HA2 40, 110, 113, and 114 was evaluated again in cleaved HA employing the p27 cleavage strategy and compared with the benchmark HA with five stabilizing substitutions in five regions of instability (Fig. 4A, Table S3).

Of note, the variant labeled AWQF in Fig. 4A, containing substitutions S40A, S110W, E113Q, and H114F, demonstrated a significant 27% improvement in trimer expression compared with our stabilized benchmark (Fig. 4A). Another variant, “ARWF” (also named “Stable_HA1HA2”), incorporated three additional substitutions (S40A, S110R, and H114F) and revealed both enhanced expression and a Tm_50_ reaching 74.8 °C. A pattern emerged suggesting that HA variants with an S110R substitution tend to exhibit greater thermal stability. These observations were confirmed through purification of both HA variants and the benchmark. The resulting Tm_50_ values were consistent with our initial screening on the cell culture supernatant (Figs. 4A and S4, Table 1). Purified Stable_HA1HA2 was subjected to mass spectrometry analysis which detected two HA1 species in a 1:1 ratio corresponding to the complete HA1 and a variant in which the C-terminal Arg was cleaved off, likely by exoproteases, confirming the occurrence of native cleavage when the RSV p27 insert is used (Figs. 4B and S5).

**Table 1.** Temperature stability of purified stabilized HA.

HA	Substitutions					*T*_onset_ (°C)^a^	Tm_50_ (°C)
	Head	Hinge	Neck	Stem	Repulsive cluster		
Benchmark	K209T	G68Y	Q64Y	H22F	E113W	64.2	71.7 ± 0.12
Stable_HA1HA2	K209T	G68Y	Q64Y	H22F, S40A, S110R, H114F	E113W	71.8	74.8 ± 0.24
AWQF	K209T	G68Y	Q64Y	H22F, S40A, S110W, H114F	E113Q	69.3	71.5 ± 0.19

^a^Mean Tm_50_ values for benchmark HA stabilized by five substitutions in five regions of instability and two variants with additional stem substitutions (Stable_HA1HA2 and AWQF). All substitutions are in HA2 except for K209T.

In subsequent tests, we examined the pH stability of purified stabilized HA proteins. By testing at different time points during 24 h incubation in buffers of varying pH levels (7.4, 5.6, 4.8, and 3.7), we found a correlation between thermostability and pH stability with the strongest differentiation of pH stability at pH 4.8 (Fig. 4C). Finally, the antigenicity profile of the purified WT and stabilized HA was assessed with biolayer interferometry which confirmed that the stabilizing substitutions did not impact antigenicity (Fig. S6).

To determine whether the stabilizing substitutions in the pH switch regions would prevent low pH triggering of HA, a critical early step in the viral life cycle, we performed cell–cell fusion experiments in HEK293 cells as described in Materials and methods. After low pH trigger, WT HA generated large syncytia and the HA with substitutions in all five regions of instability (K209T, H22F, Q64Y, G68Y, and E113W) was fully resistant to low pH-induced syncytium formation. The single-point mutation H22F in the stem switch completely prevented all syncytia formation and Q64Y and E113W markedly reduced syncytia formation (Fig. 4D).

### Cryo-EM structure of stabilized influenza B/Iowa/06/2017 HA trimer

**Fig. 5.**
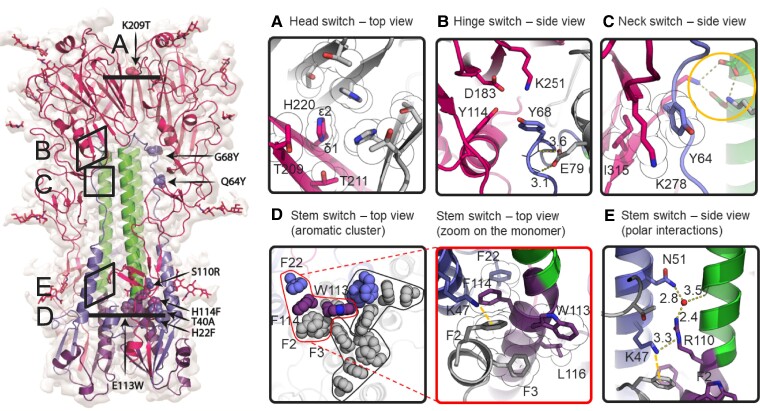
Cryo-EM analysis of stabilized pH-sensitive switch regions. A) Head switch with K209T. B) Hinge switch with G68Y. C) Neck switch with Q64Y. The orange circle indicates that polar interactions remain intact. D) Stem switch (left panel) with aromatic cluster. Interactions have been grouped into three tightly bound clusters. Side chains for one protomer are colored as in Fig. 1. (Right panel) Details of the mutations in the single interaction cluster (delimited with red line in the left panel). K47 forming the pi-cation interaction with F2 shown as orange dashed line. E) Stem switch with polar interactions with water molecule in red. The clouds around the sidechain indicate the van der Waals surface.

To better understand how these substitutions contribute to the stabilization of the prefusion HA and to assess their effect on its conformation, the structure of the natively cleaved Stable_HA1HA2 (Table 1) was determined using single particle cryo-EM to a resolution of 2.7 Å (Figs. S7 and S8, Table S4). The overall structure of the stabilized B/Iowa/06/2017 HA trimer has a root-mean-square deviation of 0.7 Å compared with the influenza B prefusion structure of B/Brisbane/60/2008 (Fig. S7) (40). All the side chains of substitutions are in the same orientation as in nonstabilized influenza B virus HA trimers from earlier studies (Fig. S7). The structure reveals that the K209T substitution mitigates potential repulsion in the head and that His220 faces upwards with Nε2, while Nδ1 is buried by Thr211 (Fig. 5). Substitutions at residues Gln64 and Gly68 in the B-loop of RR1 not only strengthen the interaction between the B-loop, the head, and the CH but also do so without disrupting other intricate polar interactions (Fig. 1). Specifically, substitution Q64Y introduces hydrophobic interactions with Lys278 and Ile315 in the head, while G68Y establishes weak polar contacts that help to shield the Glu79–backbone interprotomeric interaction from solvent. Moreover, the E113W substitution at the 3-fold axis, combined with H22F and H114F, forms an expansive hydrophobic aromatic cluster that spans the entire cross-section of the stem, involving all residues except for Phe22 in interprotomeric interactions (Fig. 5D). The S110R substitution is strategically located above the FP's Phe2 and forms polar interactions mediated by a water molecule. Concurrently, the Lys47 can serve as a proton donor to S110R, while also maintaining a pi-cation interaction with Phe2 on the other side (Fig. 5E). Overall, these substitutions collectively improve interactions and stability of the influenza B virus HA trimer by stabilization around the pH switches in the hinge and neck regions and the elimination of three pH-sensitive switches in the head and stem reducing the structure's sensitivity to changes in pH.

**Fig. 6.**
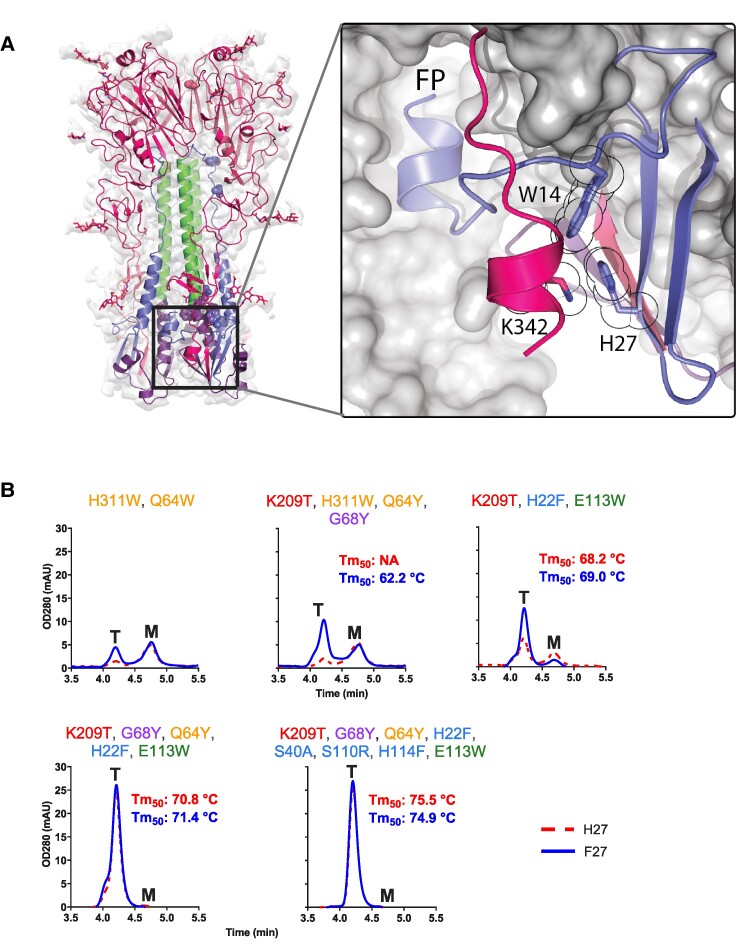
The sixth pH-sensitive switch in the HA1 C-terminal helix and intersubunit beta-sheet at the base of influenza B virus HA. A) Interacting residue and clamped FP are indicated. H27 forms a pi-cation bond with K342 and an edge-to-face aromatic interaction with neighboring W14. B) Analytical SEC of HAs with increasing number of stabilizing substitutions with H27 (red broken line) or F27 (blue solid line). Trimer (T) and monomer (M) peaks are indicated. Melting temperatures (Tm_50_) as measured by DSF in cell culture supernatant are indicated in each respective panel in red (H27) and blue (F27). Stabilizing residues are indicated above the panel and color coded as Fig. 1.

Additionally, the high-resolution structure reveals four additional residues at the HA1 C-terminus which have been disordered in all previous studied structures. Interestingly, these extra additional residues form a small C-terminal helix, which we hypothesize to be an additional pH switch composed of HA1 Lys342 and HA2 His27 in RR1 (Fig. 6A). To validate the role of His27 in this newly observed sixth pH-sensitive switch, the impact of a H27F substitution was assessed across a range of HA variants with varying stability levels (Fig. 6B). H27F substitution leads to a significant increase in trimer expression, particularly in unstable HAs. Except for the variant with eight stabilizing substitutions, an enhancement in the thermal stability was detected (Fig. 6B). Remarkably, even for the variant that already contained eight stabilizing substitutions, the addition of H27F improved the pH stability at pH 3.7 and pH 4.8 (Fig. 4C), identifying this pH switch as the sixth, untargeted area of instability in our prior protein engineering efforts. Next, the structure of the natively cleaved *Stable_HA1HA2* was solved at pH 5.5 using cryo-EM. A structural comparison with the neutral pH structure showed that at low pH, the base of the HA trimer becomes disordered and no density was observed around the sixth pH-sensitive switch composed of the small HA1 C-terminal helix that contains Lys342 and the antiparallel beta-sheet that contains HA2 His27 (Figs. S10 and S11).

## Discussion

Influenza causes a considerable health and economic burden, and the most effective means of preventing seasonal influenza virus infections are through annual vaccination. Improvement of these conventional vaccines is important because the effectiveness is variable from year to year (41–43). Recent advances in vaccinology, particularly the rise of RNA- and particle-based platforms, have redefined approaches to vaccine development. For both traditional and emerging vaccine development strategies, the expression of high-quality antigens in their correct prefusion conformation is pivotal. Stabilizing the prefusion state of fusion proteins has proven to be crucial for the induction of efficacious immune responses, marking a grand challenge in the field of structural vaccinology (5, 7, 44–46). Understanding the refolding mechanism of class I fusion proteins and identifying their unstable regions are keys for the development of high-quality immunogens. For influenza HA, refolding is initiated at lower pH levels, but even at neutral pH, the HA is unstable and the soluble ectodomain is not fully trimeric (Figs. 2 and S2) (28). In previous work on influenza A, we found that three substitutions in two pH switches enhanced the quaternary structure and impeded refolding of HA (28). Although the pH switches between influenza A groups 1 and 2 are conserved, this is not the case with influenza B virus HA, despite the high structural homology. This discrepancy is likely due to the larger trimer interface in influenza B compared with influenza A (6,931 vs. 5,474 Å^2^, Fig. S12), necessitating more pH switches for refolding and fusion. In the current study, we have analyzed diverse pH switches, distributed throughout the entire ectodomain, in which the protonated histidines play either a repulsive or an attractive role. While pH-sensitive switches have evolved independently in influenza A and B, switches in the stem occupy an equivalent location. Both influenza A and B HAs have a switch at the interface of the kink between the central helices C and D. In the case of influenza B virus HA, this region is destabilized by protonation of His114 and His22—reminiscent of pH-switch 2 in influenza A HA (Fig. 1 (28)). Additionally, His22 is part of the intersubunit beta-sheet composed of the N-terminus of HA1 and the RR1 and RR2 of HA2. Even though its location is higher in the stem of influenza B compared with influenza A, its’ protonation still destabilizes the intersubunit beta-sheet, much like pH-switch 1 in influenza A (28). Interestingly, substitutions in the stem pH-switch and repulsive cluster of Glu113 had the strongest impact on fusion (Fig. 4D) and are the only elements also conserved in the sister clade of influenza B, as indicated by the recently described Wuhan spiny eel influenza virus (47). Our structural analysis also revealed a novel sixth pH switch, facilitated by the natively cleaved HA1 C-terminal helix that stabilizes influenza B virus HA by clamping the FP's proximal region (Fig. 6). The complete release of the FP depends on both the previously described stem switch and this new sixth pH switch in the intersubunit beta-sheet.

This study provides an in-depth analysis and evaluation of six pH-sensitive switches in influenza B virus HA. It also marks the first discovery of stabilizing substitutions near or within these pH switches, which have been shown to enhance trimer expression levels, stability, and quaternary structure. When these stabilizing substitutions are combined, they not only further improve the expression and quality, but also eliminate the pH sensitivity of HA. This enables cleavage of HA upon passage through the exocytic pathway, leading to a cleaved and fully closed HA trimer with a melting temperature ∼15 °C higher than its uncleaved WT counterpart. Conventionally, cleavage of HA0 is accomplished by addition of exogenous trypsin, a method that can be difficult to regulate, potentially leading to incomplete cleavage and an undesired, nonnative HA1 C-terminus. Incomplete cleavage significantly impacts the stability and antigenic structure of the HA stem. For instance, an uncleaved influenza B virus HA trimer displays a splayed base, exposes the FP on the protomer surface, and prevents the HA1 C-terminus from adopting the helical structure that clamps the FP (Fig. S1). Inadequate cleavage of influenza B virus HA remains a significant challenge for many existing commercial influenza vaccines (30). It may also have contributed to the suboptimal performance of the influenza B component in a recent Ph1 mRNA trial (48). As such, the “repair, stabilize, and cleave” strategy described in this study holds promise for improving the design of future influenza B virus HA-based vaccines and therapeutics. The study shows that structural vaccinology not only paves the way for developing new vaccines against pathogens such as RSV, human metapneumovirus, or coronavirus (5, 7, 44, 46, 49, 50), but also optimizes the expression, quality, and stability of natively folded, closed HA1/2 heterotrimers and holds promise for improved next-generation influenza vaccines.

## Materials and methods

### Expression of HA proteins

DNA fragments encoding C-tagged HA proteins which were C-terminally extended with a four residue affinity tag EPEA (C-tag) were synthesized (Genscript) and cloned in the pcDNA2004 expression vector, a modified pcDNA3 plasmid with an enhanced cytomegalovirus promotor. Culture supernatants for analytical SEC and DSF were generated by transient transfection of Expi293F cells at 200 µL scale in 96-half deep well plates at a cell density of 2.5E + 06vc/mL using the ExpiFectamine 293 transfection kit (Gibco, Thermo Fisher Scientific). At day 3, posttransfection culture supernatants were harvested, clarified by centrifugation (10 m at 400 × *g*) and filtered (96-well filter plates, 0.22 µm polyvinylidene difluoride membrane; Corning). Protein batches generated for purification were produced in Expi293F suspension cells (70 mL scale). Expi293F cells were cultured in Expi293F Expression medium [+] GlutaMAX (Gibco, Thermo Fisher Scientific) and transiently transfected using ExpiFectamine 293 (Gibco, Thermo Fisher Scientific) according to the manufacturer's instructions, and 18 h posttransfection enhancers 1 and 2 were added (Gibco, Thermo Fisher Scientific). Culture supernatants were harvested at day and were clarified by centrifugation, followed by filtration over a 0.2-µm bottle top filter (Corning).

### Purification of HA proteins

From the harvested culture supernatants, HA proteins were purified by a two-step protocol using and ÄKTA Avant 25 system (GE Healthcare Life Sciences). Clarified supernatant was loaded on a C-tag XL 5 mL prepacked column (Thermo Fisher Scientific) equilibrated in 20 mM tris(hydroxymethyl)aminomethane hydrochloride, 150 mM NaCl, pH 7.4. Elution of the C-tagged proteins was performed using an equilibration buffer containing 2 M MgCl_2_. To further polish the purified protein, SEC was performed by running a HiLoad Superdex 200 16/600 column (GE Healthcare Life Sciences). Peak fractions were pooled and sterile filtrated using a Millex-GV 0.22 µM filter membrane (Millipore Sigma).

### Size exclusion chromatography and multiangle light scattering

Expi293F cell culture harvests were analyzed for the presence of expressed HA, and purity of produced proteins was analyzed by analytical SEC, as described previously (28). An ultra high-performance liquid chromatography Vanquish system (Thermo Fisher Scientific) in combination with a Unix-C SEC-300 column (Sepax Technologies Inc.) and an in-line µDAWN instrument (Wyatt Technology), µT-rEx differential refractometer (Wyatt Technology), and Nanostar DLS reader (Wyatt Technology) were run in 150 mM sodium phosphate, 50 mM sodium chloride, pH 7.0. The UV signal of supernatants of nontransfected cells was subtracted from the UV signal of HA transfected cells. SEC profiles were analyzed by the Astra software package (Wyatt Technologies), and molecular weight calculations were derived from the light scattering and refractive index signal using a d*n*/d*c* value of 0.185. Mock transfections without the addition of plasmid were used to perform baseline corrections in analytical SEC.

### Differential scanning fluorimetry

The stability of the HA proteins in supernatant was determined by measuring the melting temperature (Tm) using DSF. Sypro Orange Dye 5000× (Invitrogen) was diluted in phosphate-buffered saline (PBS; 1:250) to obtain a 20× working solution. For each reaction, 10 µL of the supernatant was mixed with 3 µL of the Sypro 20× and 17 µL of PBS in a MicroAmp Fast Optical 96-well plate (Thermo Fisher Scientific). For measuring the melting temperature of purified proteins, Sypro 20× was added to 6 µg HA protein in solution. PBS was used as a negative control. The plate was covered with a MicroAmp Optical Adhesive Film (Thermo Fisher Scientific) and was subsequently read in a ViiA7 real-time PCR machine. Melting temperature for all HA variant was expressed as the temperature in which 50% of the protein was melted (Tm_50_). The measurements were performed with a starting temperature of 25 °C and a final temperature of 95 °C (54 °C increase per hour). Melting curves were measured using a ViiA7 real-time PCR machine (Applied Biosystems), and Tm_50_ values were derived from the negative first derivative. Mock transfections without the addition of plasmid were used to perform baseline corrections in DSF.

### Gel electrophoresis

Samples were prepared by adding 4 µL of 4× LDS buffer (Invitrogen) to 12 µL of supernatant. Samples were heated for 10 min at 95 °C without shaking in a Thermomixer (Eppendorf). From a NuPAGE 4 to 12%, Bis-Tris, 1.0 mm, 12 wells (Thermo Fisher Scientific), the comb and white sticker were removed and rinsed with demi water prior to being placed in a bolt gel running system (Thermo Fisher Scientific). The inner and outer chambers were filled with NuPAGE MES SDS Running Buffer (20X; Thermo Fisher Scientific), and 7 µL of sample and 5 µL PageRuler Plus Prestained Protein Ladder (Thermo Fisher Scientific) were loaded. A powerpack basic (Bio-Rad) was connected, and the gel was run at 200 V for 30 min. After running, the gel was stained with InstanBlue Coomassie Protein Stain (Abcam, ab119211) for 1 h before a picture was taken using an Odyssey M (Li-Cor).

### Biolayer interferometry using monoclonal antibodies

Sensors were rehydrated for, at least, 15 min before use. Antibodies at a concentration of 10 μg/mL in 1× kinetics buffer (Sartorius, cat. #18-1105) were immobilized to anti-hIgG sensors (FortéBio, cat. #18-5060) using 96-well black flat-bottom polypropylene microplates (Corning, cat. #3694) in a 10-min step, using an Octet RED384 system (FortéBio) with a shaking speed of 1,000 rpm at 30 °C. A baseline was set in 1× kinetic buffer for 60 s, the antibody immobilized for 600 s followed by a baseline in 1× kinetic buffer for 150 s. Association with the influenza virus HA protein, at 10 μg/mL in 1× kinetics buffer, was performed for 600 s followed by dissociation phase in 1× kinetic buffer of 600 s. Data analysis was performed using FortéBio Data Analysis 12.0 software (FortéBio).

### Cell–cell fusion assay

To test the effect of stabilization of the pH switch regions on HA fusogenicity, we performed cell–cell fusion experiments. To this end, plasmids encoding full-length WT HA or stabilized variants thereof, human TMPRSS2 and mScarlet were co-expressed from pcDNA2004 plasmids in HEK293 cells using Trans-IT transfection reagent according to the manufacturer's instructions. Transfections were performed on 80% confluent cell monolayers in 24-well plates. After 24 h incubation at 37 °C and 10% CO_2_, the transfected cells were exposed to pH 5.0 DMEM for 10 min at 37 °C, after which the medium was aspirated and replaced with normal culture medium. The cells were incubated for an additional 1 h at 37 °C in the presence of Hoechst to allow the cytoskeleton rearrangements involved in syncytia formation before being imaged using an EVOS cell imaging system (Thermo Fisher Scientific). Overlays between red and blue channels were made in ImageJ.

### Preparation of grid and data acquisition

Purified influenza B virus HA ternary complex (3.5 µL) was applied to the plasma-cleaned (Gatan Solarus) Quantifoil 1.2/1.3 UltraAuFoil holey gold grid, which was then vitrified using a Vitrobot Mark IV (FEI Company). The cryogrids were loaded into a Glacios transmission electron microscope (Thermo Fisher Scientific) operating in nanoprobe at 200 keV with a Falcon IV direct electron detector. Images were recorded with EPU (Thermo Fisher Scientific) in counting mode with a pixel size of 0.948 Å and a nominal defocus range of −2.4 to −0.8 μm. Data collection occurred at a dose rate of 5.3 electrons per physical pixel per second, and images were recorded with a 7.5-s exposure in electron-event representation format corresponding to a total dose of 40.0 electrons per Å^2^. All details corresponding to individual datasets are summarized in Table S3.

### EM data processing

A total of 5,974 movies were collected for the influenza B virus HA at neutral pH. The movies underwent beam-induced motion correction, estimation of contrast transfer function parameters, automated reference particle picking, and particle extraction with a box size of 280 pixels. Two-dimensional classification was performed in CryoSPARC (51) live during the data acquisition. Particle images showing clear HA features were merged and subjected to ab initio 3D reconstruction with C3 symmetry in CryoSPARC. Several rounds of optimized 3D heterogeneous refinement resulted in 2 classes with distinct HA density, containing a total of 481,814 particles. These particles underwent refinement using nonuniform and local refinements within CryoSPARC with C3 symmetry. Local resolution was calculated using ResMap (52). Resolution estimates were based on the gold-standard Fourier shell correlation (FSC) = 0.143 criterion, applying a soft mask around the protein complex density. Density maps were sharpened using different negative temperature factors via automated procedures. Half maps were used for model building. Details regarding the number of particles in each dataset and other processing steps are summarized in Fig. S7 and Table S3. The influenza B virus HA cryo-EM structure at low pH (5.5) was determined using a similar protocol, except for a 1-h dialysis in the low pH buffer before loading onto EM grids (Fig S9, Table S3).

### Model building and refinement

The initial template of the influenza B virus HA was generated from a homology-based model calculated by SWISS-MODEL (53). This model was then docked into the EM density map using Chimera (54), followed by manual adjustment using COOT (55). Each model was independently refined and minimized in real space using the *phenix.real_space_refine* module in PHENIX (56) against separate EM half maps with default parameters. The model was refined into a working half map, and model improvement was monitored using the free half map. The geometry parameters of the final models were validated using COOT, MolProbity (57), and EMRinger (58). Refinements were conducted iteratively until no further improvements were observed. Final refinement statistics are provided in Table S3. Model overfitting was assessed by refining against one cryo-EM half map, and FSC curves were calculated between the resulting model and the working half map, as well as between the resulting model and the free half and full maps for cross-validation. Figures were generated using PyMOL (The Pymol Molecular Graphics System, Version 2.0 Schrödinger, LLC) and Chimera (54).

## Supplementary Material

pgae462_Supplementary_Data

## Data Availability

The maps for the neutral and low pH structures were deposited in the Electron Microscopy Data Bank with IDs EMD-42060 and EMD-43273, respectively. The atomic model for the neutral pH HA trimer is available in the Protein Data Bank with ID 8UAD. Mass spectrometry data can be accessed via ProteomeXchange with identifier PXD046130.
