# Supplementary material for: Engineering a cleaved, prefusion-stabilized influenza B virus hemagglutinin by identification and locking of all six pH switches
Source: PNAS Nexus. 2024 Oct 11;3(10):pgae462. doi: 10.1093/pnasnexus/pgae462 (PMC11497598; doi:10.1093/pnasnexus/pgae462)
Supplement: pgae462_Supplementary_Data [file pgae462_supplementary_data.zip › PNASNEXUS-PNASNEXUS-2024-00816-TR-s02.pdf]

**Title:**

# Engineering a cleaved, prefusion-stabilized influenza virus B hemagglutinin by identification and locking of all six pH-switches


**Authors**

Jarek Juraszek[1]*, Fin J. Milder[1]*, Xiaodi Yu[2], Sven Blokland[1], Daan van Overveld[1], Pravien Abeywickrema[2], Sem Tamara[1], Sujata Sharma[2], Lucy Rutten[1], Mark J. G. Bakkers[1,3], Johannes P.M Langedijk[1,3#]

**Affiliations:**

[1] Janssen Vaccines & Prevention BV, Leiden, The Netherlands

[2] Structural & Protein Science, Janssen Research and Development, Spring House, PA 19044, USA

[3] Current affiliation: ForgeBio, Amsterdam, The Netherlands

* Authors contributed equally

#Correspondence to: hlangedijk@forge-bio.com

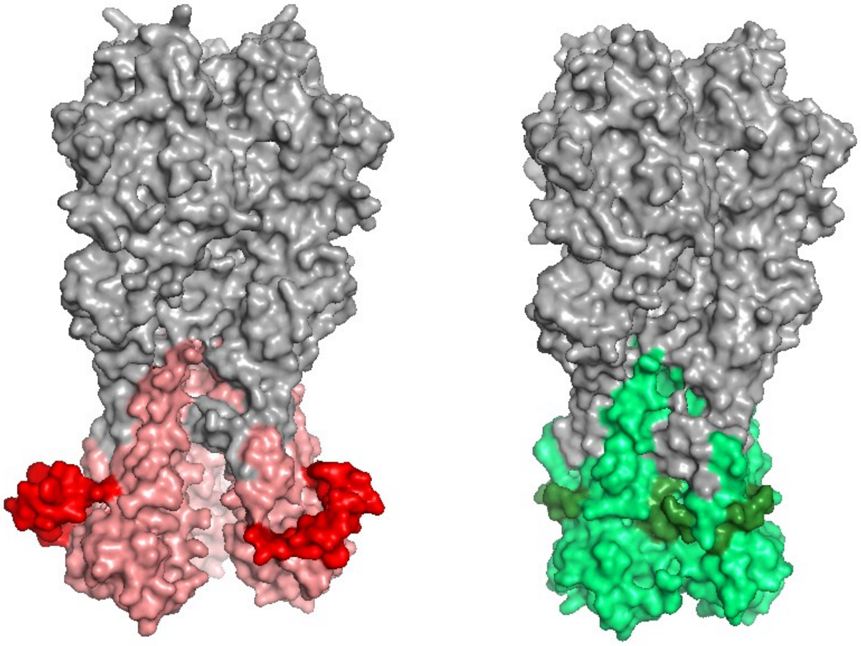

**Fig. S1. Surface representation of structure of uncleaved (6CNV, left) and cleaved (F4QM, right) influenza B virus HA trimer.** Stem regions are colored red for the splayed uncleaved and green for the cleaved HA. Darker colors correspond to the fusion peptide and fusion peptide proximal region which are in different locations for both HAs (residues 339-367). For 6CNV the missing part of the fusion peptide residues 348-367 was modeled using SWISS-MODEL.

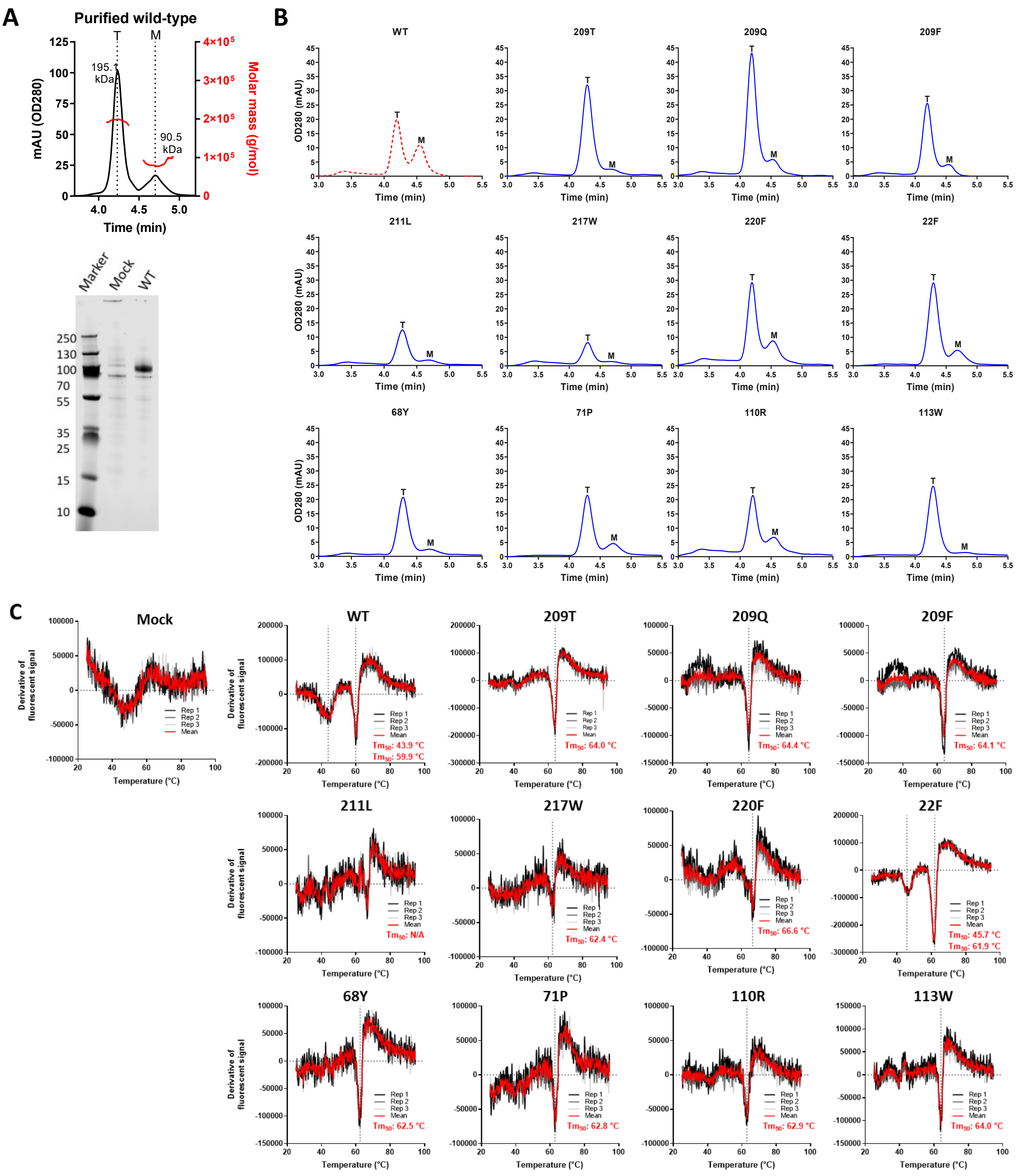


**Fig. S2. Characterization of stabilized HA in cell culture supernatant.** (A) Size exclusion chromatography, multi-angle light scattering (SEC-MALS) trace of analysis of purified HA. The molar mass as determined by MALS at peak max of the trimer and monomer indicated (top) and gel electrophoreses of supernatant of mock and wt HA transfected cells (bottom). (B) Analytical SEC profiles and (C) Melting curves of clarified supernatant of Expi293F cells expressing B/Iowa/06/2017 HA variants. Comparisons were tested in the same screen on the same day and the reproducibility of transfections and analysis were ensured by including a control for each transfection series. Baseline corrections are performed on SEC and melting curves by subtraction of mock signal.

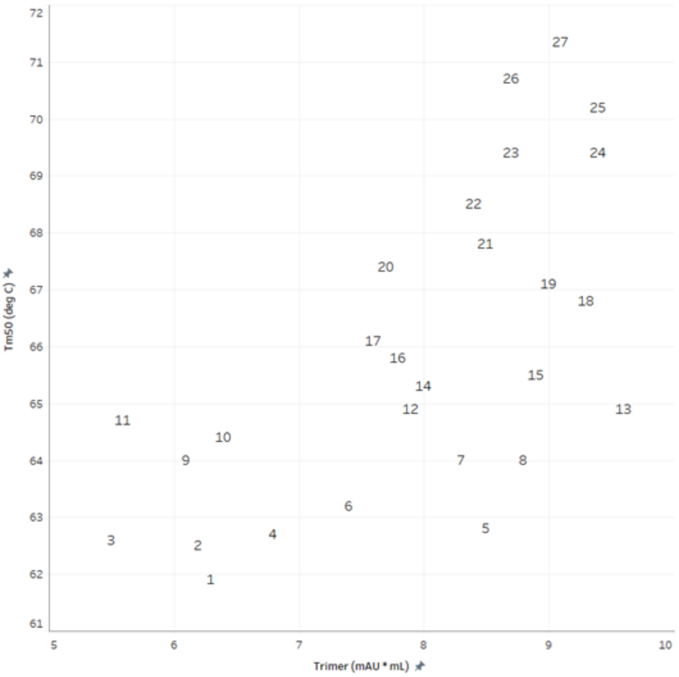


| | | Head switch | | | Hinge loop | | Neck switch | Repulsive cluster | Stem switch | |
|---|---|---|---|---|---|---|---|---|---|---|
| | | 209 | 211 | 218 | 68 | 217 | 64 | 113 | 22 | 114 |
| Index | Mutation list | K | T | T | G | V | Q | E | H | H |
| 1 | K209T+T218N | T | | N | | | | | | |
| 2 | K209T+T211L | T | L | | | | | | | |
| 3 | K209T+V217K | T | | | | K | | | | |
| 4 | G68Y+Q64F | | | | Y | | F | | | |
| 5 | G68Y+Q64Y | | | | Y | | Y | | | |
| 6 | E113Q+H22F | | | | | | | Q | F | |
| 7 | E113Q+Q64Y | | | | | | Y | Q | | |
| 8 | E113W+Q64Y | | | | | | Y | W | | |
| 9 | K209L+T211L | L | L | | | | | | | |
| 10 | E113Q+G68Y | | | | Y | | | Q | | |
| 11 | E113W+H114F | | | | | | | W | | F |
| 12 | E113W+H22F | | | | | | | W | F | |
| 13 | K209T+Q64Y | T | | | | | Y | | | |
| 14 | H22F+K209T | T | | | | | | | F | |
| 15 | G68Y+K209T+Q64Y | T | | | Y | | Y | | | |
| 16 | E113Q+K209T | T | | | | | | Q | | |
| 17 | G68Y+K209T | T | | | Y | | | | | |
| 18 | H22F+K209T+Q64Y | T | | | | | Y | | F | |
| 19 | E113Q+K209T+Q64Y | T | | | | | Y | Q | | |
| 20 | E113W+K209T | T | | | | | | W | | |
| 21 | E113Q+G68Y+K209T+Q64Y | T | | | Y | | Y | Q | | |
| 22 | E113Q+H22F+K209T+Q64Y | T | | | | | Y | Q | F | |
| 23 | E113Q+G68Y+H22F+K209T+Q64Y | T | | | Y | | Y | Q | F | |
| 24 | E113W+K209T+Q64Y | T | | | | | Y | W | | |
| 25 | E113W+G68Y+K209T+Q64Y | T | | | Y | | Y | W | | |
| 26 | E113W+H22F+K209T+Q64Y | T | | | | | Y | W | F | |
| 27 | E113W+G68Y+H22F+K209T+Q64Y | T | | | Y | | Y | W | F | |

**Fig. S3. Reference plot and Table for identification markers in figure 2 B, right panel**

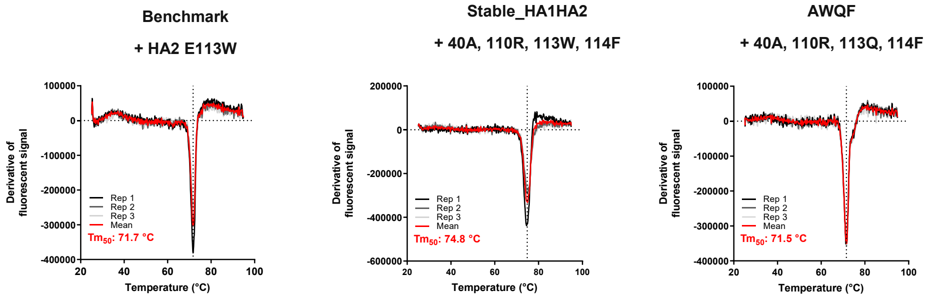


**Figure S4. Temperature stability of purified stabilized HA.** Analysis of melting temperature using differential scanning fluorimetry. The first order derivatives are plotted. Shown are the triplicate runs with the averaged highlighted in red. Tm is determined as the lowest derivative value representing the $Tm_{50}$ value. All HAs contain HA1 K209T, HA2 H22F, Q64Y, G68Y) and additional stabilizing substitutions are indicated. Left panel is benchmark with 5 substitutions in 5 regions of instability, middle panel is Stable_HA1HA2 labeled ARWF in Figure 4 and right panel is labeled AWQF in Figure 4.

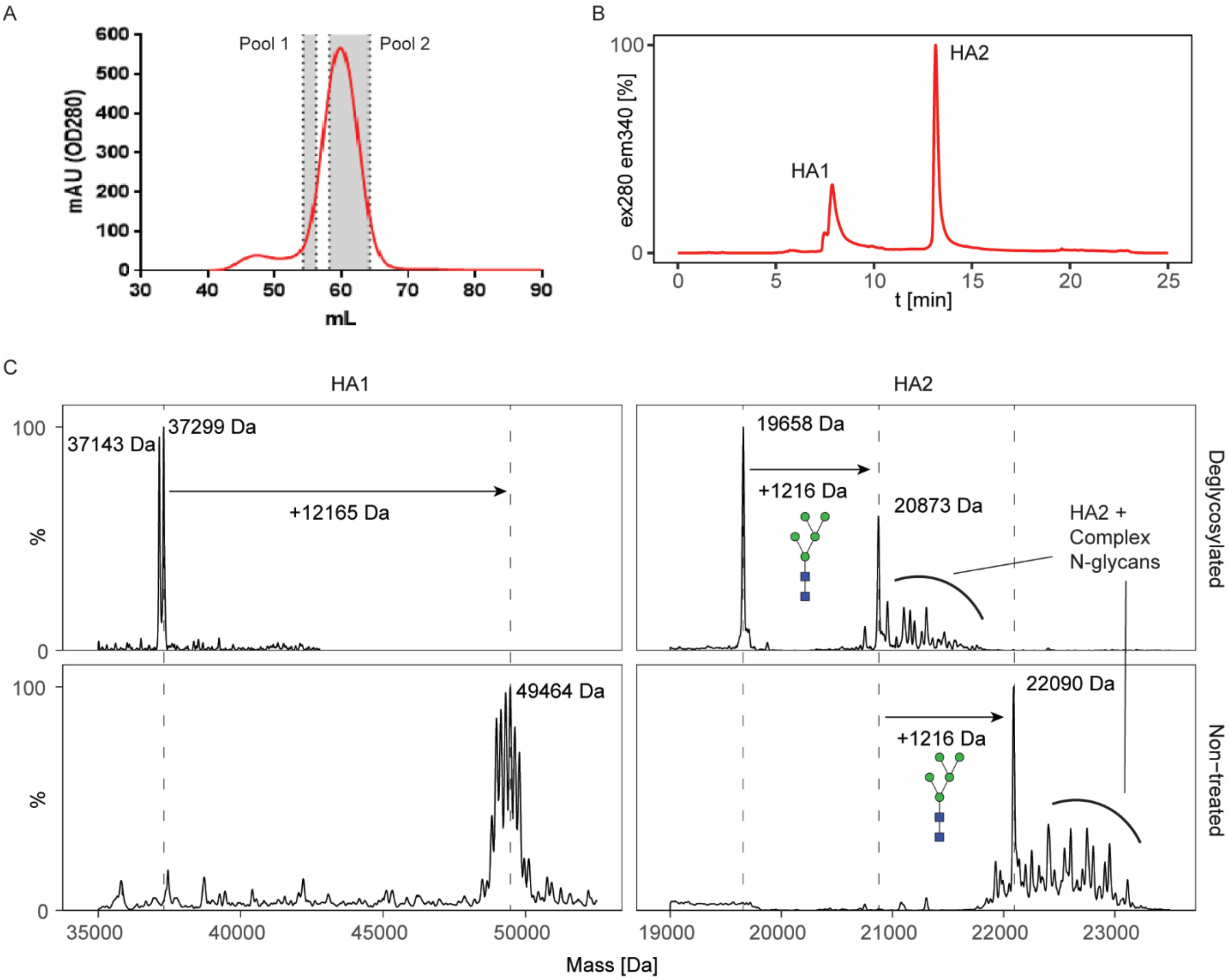


D

| Condition | Component | Proteoform | Observed mass [Da] | Expected mass [Da] | Difference [Da] | ppm | Fractional abundance [%] |
|---|---|---|---|---|---|---|---|
| Deglycosylated | HA1a | Fully cleaved (DRI...LKE) | 37143 | 37143.3 | -0.3 | -9 | 49 |
| | HA1b | Additional Arg at C-term (DRI...LKER) | 37299 | 37299.5 | -0.5 | -13 | 51 |
| | HA2 | Fully cleaved (GFF...PEA) | 19658 | 19658.2 | -0.2 | -8 | 41 |

**Fig. S5. Intact mass LC-MS analysis of HA1 and HA2 chains from SEC purification**. (A) SEC profile with fractions collected for the liquid chromatography-mass spectrometry (LC-MS) analysis annotated. Panels B-D show data for pooled fraction. (B) LC chromatogram whereby fluorescence signal (excitation 280 nm, emission 340 nm) reveals two peaks corresponding to HA1 and HA2 chains. (C) Deconvoluted mass profiles for the HA1 (left) and HA2 (right) proteins under reducing and deglycosylating conditions (top row) and only reducing conditions (bottom row). Masses of the major peaks in the spectra and mass shifts between the two conditions are annotated. (D) Table with differences between experimental and theoretical masses of HA backbones.

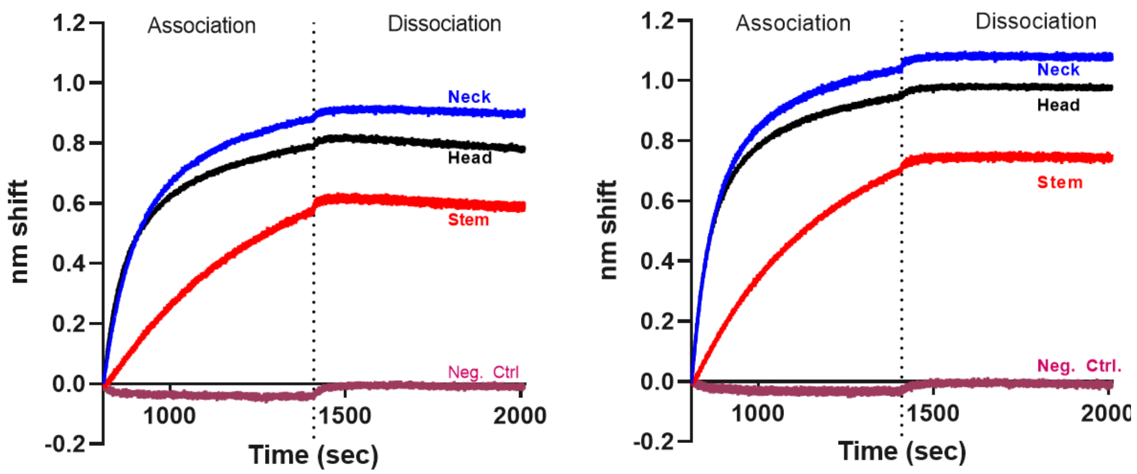


**Fig. S6. Antigenicity of WT and stabilized HA.** BLI using kinetic Octet with immobilized mAbs SD84 against the head (black), CR8071 against the neck (blue), SD83 against the stem (red) and negative control PGT145 against HIV1 Env[35 38], followed subsequently by purified wt HA (left) and stabilized HA (right) of purified influenza B/Iowa/06/2017, followed by kinetic buffer during dissociation phase.

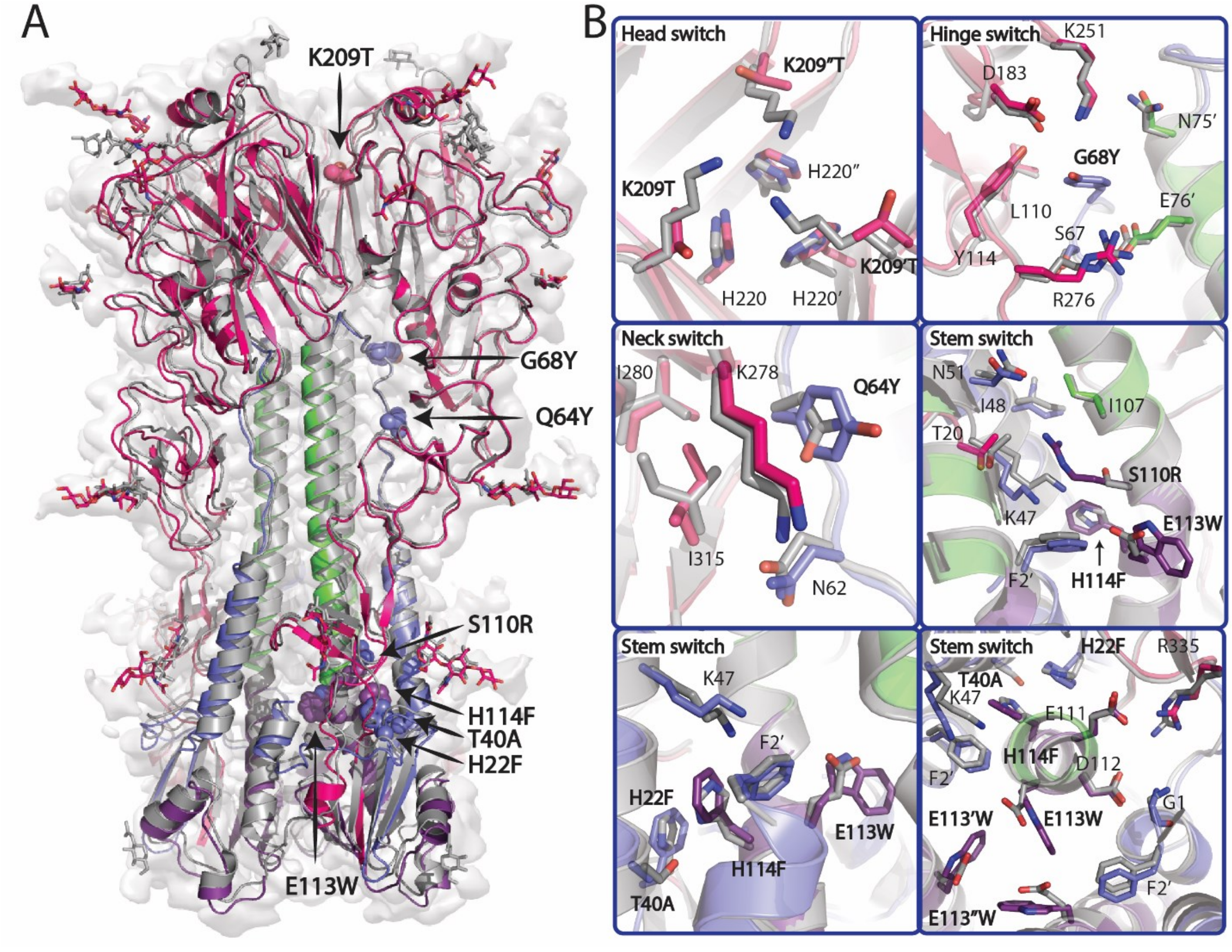


**Fig. S7. Structural comparison of stabilized and WT HA.** (A) Global structural comparison of stabilized (colored as in Fig. 1A) and WT (PDBID: 4m44, colored in gray) influenza B virus HA protein in prefusion, with gray EM density surface. Stabilization mutations were highlighted. (B) Zoomed-in views of pH-sensitive switch regions (Head, Hinge, Neck, and Stem) in stabilized and WT influenza B virus HA protein structures. The side chains of key residues were shown as sticks.

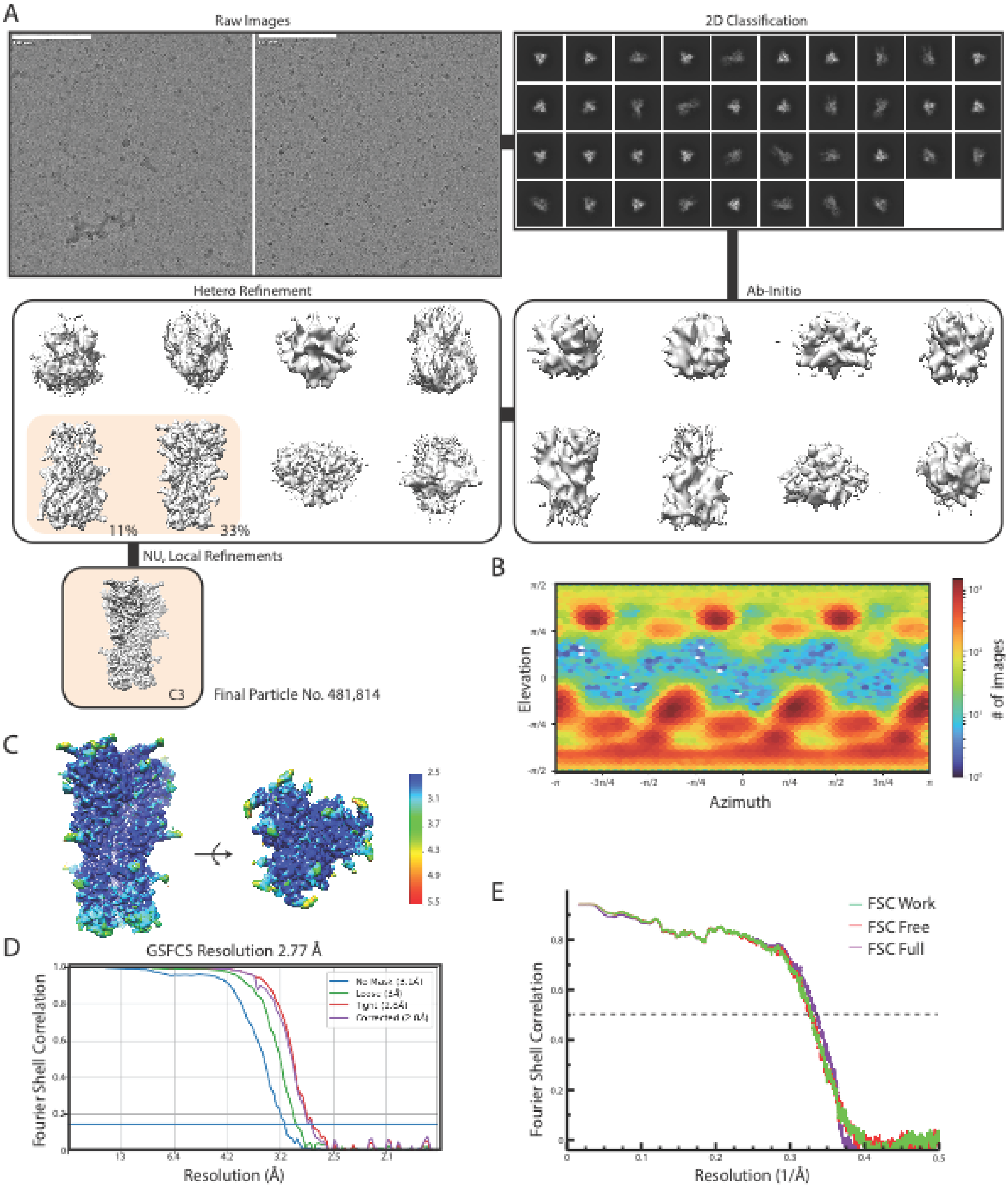


**Fig. S8. Cryo-EM analysis of influenza B virus HA ternary complex at neutral pH.** (A). Flow chart of the cryo-EM data processing procedure. Details can be found in the Materials and Methods. (B) Angular orientation distribution of the particles used in the final reconstruction. The particle distribution is indicated by different color shades. (C) Local resolution of the map and colored as indicated. (D). Fourier shell correlation (FSC) curves of the structure with FSC as a function of resolution using CryoSPARC Local refinement outputs. (E). Comparison of the FSC curves between model and half map 1 (work), model and half map 2 (free), and model and full map are plotted in green, red, and magenta, respectively.

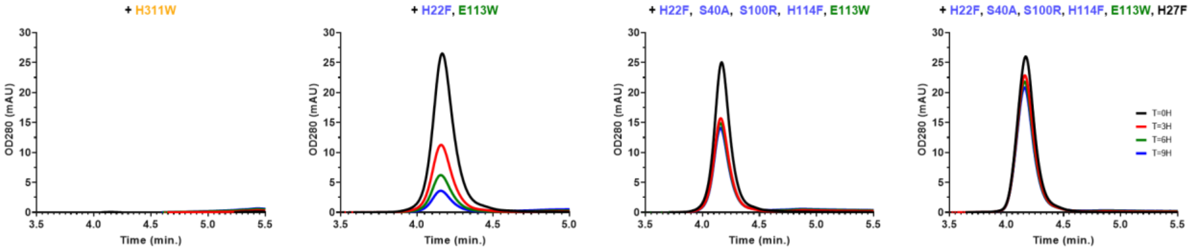


**Fig. S9. pH stability of HA variants described in Figure 4C.** Analytical SEC of HA variants incubated at pH 4.8 for 9h. Variants contain increasing amount of stabilizing substitutions. All variants contain K209T, G68Y, Q64Y and substitution indicated above panel. Substitutions are indicated and are color coded as in Fig. 1.

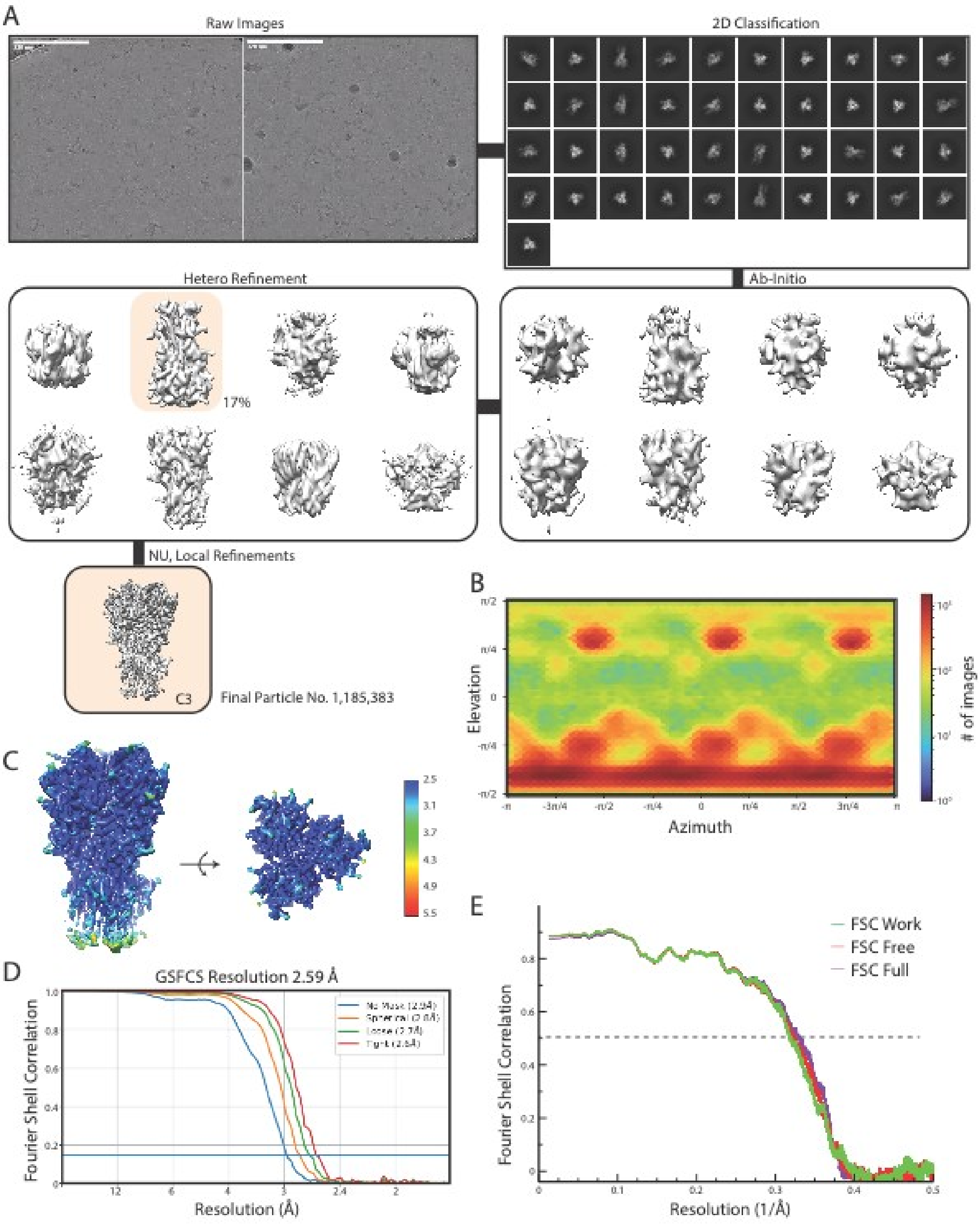


**Fig. S10. Cryo-EM analysis of influenza B virus HA ternary complex at low pH.** (A). Flow chart of the cryo-EM data processing procedure. Details can be found in the Materials and Methods. (B) Angular orientation distribution of the particles used in the final reconstruction. The particle distribution is indicated by different color shades. (C) Local resolution of the map and colored as indicated. (D). Fourier shell correlation (FSC) curves of the structure with FSC as a function of resolution using CryoSPARC Local refinement outputs. (E). Comparison of the FSC curves between model and half map 1 (work), model and half map 2 (free), and model and full map are plotted in green, red, and magenta, respectively.

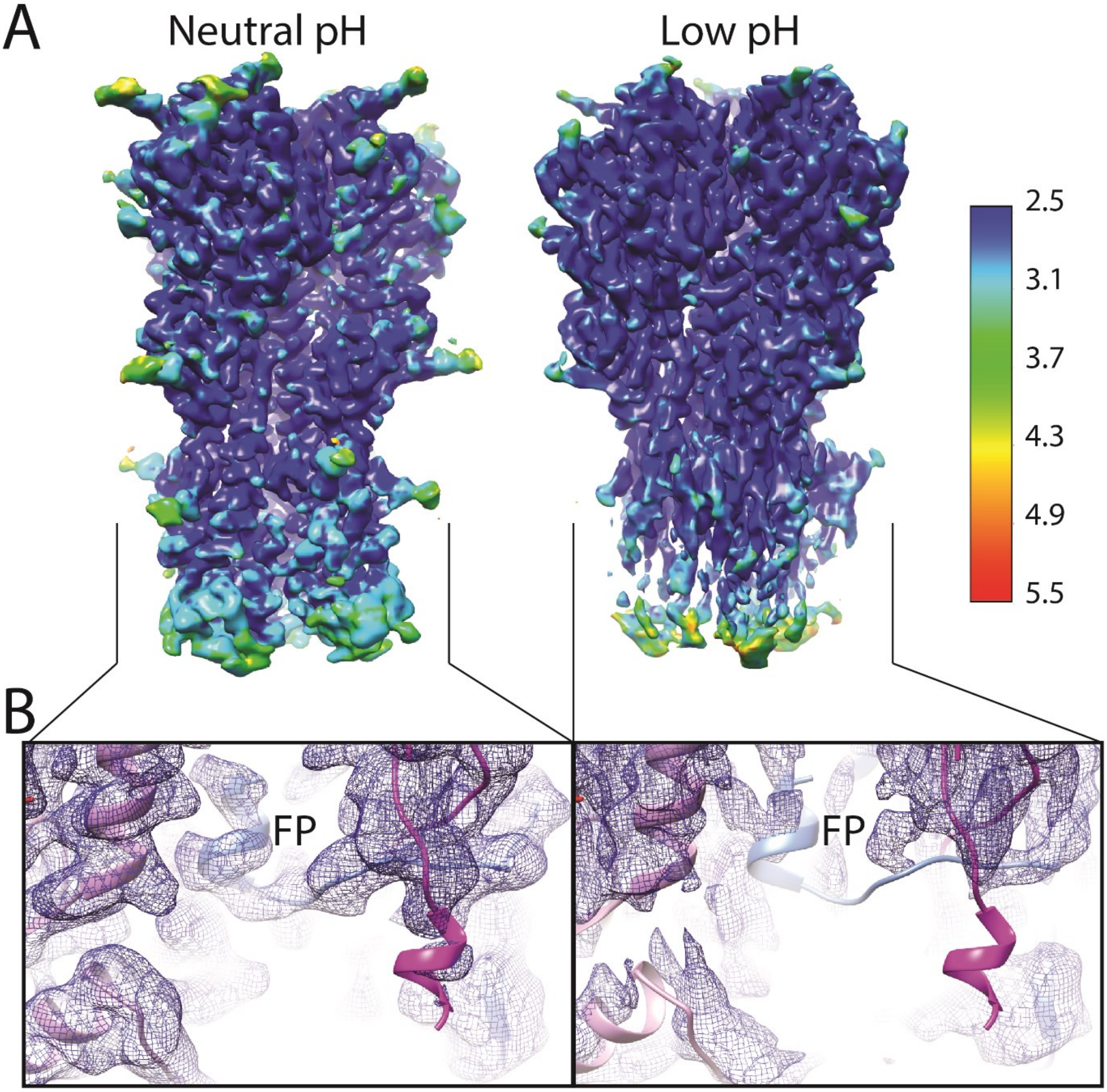


**Fig. S11. Cryo-EM maps of stabilized influenza B virus HA at neutral and low pH.** (A) Electron density maps of cleaved Stable_HA1HA2 at pH 7.4 (left) and pH 5.5 (right) with color coding of local resolutions, and (B) panels with detailed zoomed-in view on the vicinity of the sixth pH switch showing loss of density for HA1 C-terminal helix (red).

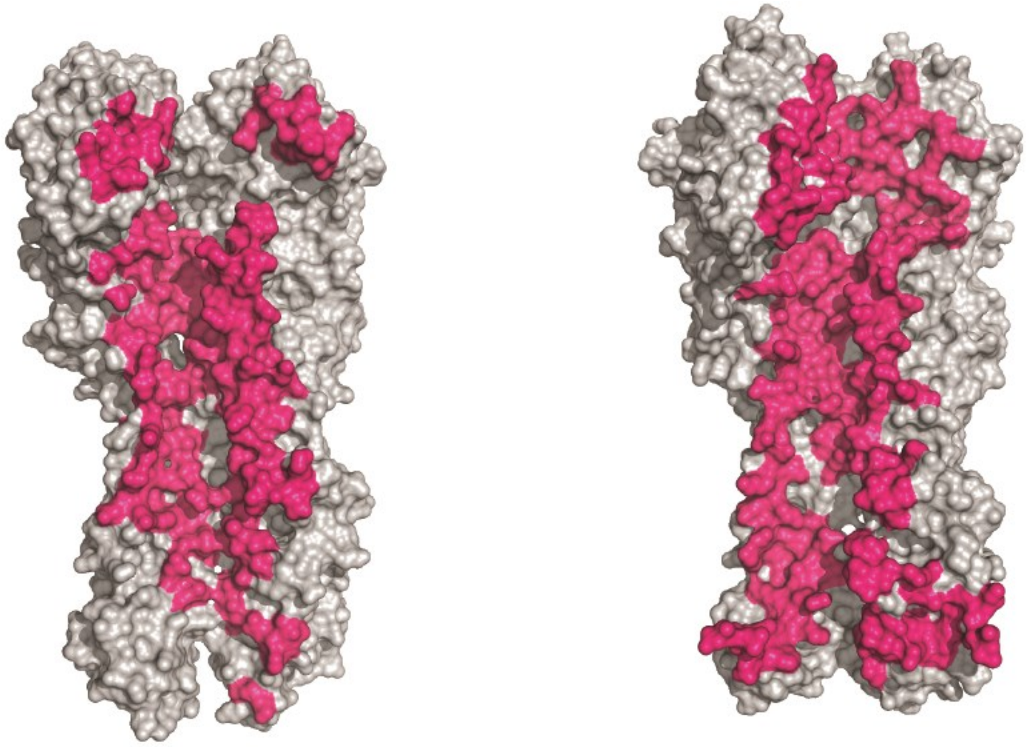

**Fig. S12. HA trimer interface of influenza A and B virus.** type A (left, 3UBQ) and B (right, 4M44) showing only two protomers of the HA trimer with the trimer interface in pink.

**Table S1. Melting temperatures of stabilized HA in cell culture supernatant.**

| | Head switch | | | | | | Hinge loop | | | | Neck switch | | | | Repulsive cluster | | Stem Switch | | | | | Base | Fusion peptide | | | | Receptor binding site | Temperature stability $Tm_{50}$ (°C) |
|---|---|---|---|---|---|---|---|---|---|---|---|---|---|---|---|---|---|---|---|---|---|---|---|---|---|---|---|---|
| Mutation list | 209K | 211T | 213S | 218T | 220H | 259T | 217V | 67S | 68G | 71D | 311H | 64Q | 91T | 93A | 113E | 116L | 22H | 40S | 110S | 111E | 114H | 30H | 11E | 15E | 18I | 29A | F152 | |
| WT | | | | | | | | | | | | | | | | | | | | | | | | | | | | 60.1 |
| F152W | | | | | | | | | | | | | | | | | | | | | | | | | | | W | 61.1 |
| K209L | L | | | | | | | | | | | | | | | | | | | | | | | | | | | 63.3 |
| K209T | T | | | | | | | | | | | | | | | | | | | | | | | | | | | 63.9 |
| K209R | R | | | | | | | | | | | | | | | | | | | | | | | | | | | 60.2 |
| K209Q | Q | | | | | | | | | | | | | | | | | | | | | | | | | | | 64.4 |
| K209F | F | | | | | | | | | | | | | | | | | | | | | | | | | | | 64.1 |
| K209I | I | | | | | | | | | | | | | | | | | | | | | | | | | | | 63.9 |
| K209Y | Y | | | | | | | | | | | | | | | | | | | | | | | | | | | 62.9 |
| T211V | | V | | | | | | | | | | | | | | | | | | | | | | | | | | 57.5 |
| T211L | | L | | | | | | | | | | | | | | | | | | | | | | | | | | 66.7 |
| S213A | | | A | | | | | | | | | | | | | | | | | | | | | | | | | 59.9 |
| V217W | | | | | | | W | | | | | | | | | | | | | | | | | | | | | 62.4 |
| V217K | | | | | | | K | | | | | | | | | | | | | | | | | | | | | 59.2 |
| T218V | | | | V | | | | | | | | | | | | | | | | | | | | | | | | 58.8 |
| T218N | | | | N | | | | | | | | | | | | | | | | | | | | | | | | 59.1 |
| T218I | | | | I | | | | | | | | | | | | | | | | | | | | | | | | 59.0 |
| H220T | | | | | T | | | | | | | | | | | | | | | | | | | | | | | 59.1 |
| H220T | | | | | N | | | | | | | | | | | | | | | | | | | | | | | 57.9 |
| H220T | | | | | Q | | | | | | | | | | | | | | | | | | | | | | | 56.8 |
| H220T | | | | | F | | | | | | | | | | | | | | | | | | | | | | | 66.6 |
| T259V | | | | | | V | | | | | | | | | | | | | | | | | | | | | | 60.0 |
| H311M | | | | | | | | | | | M | | | | | | | | | | | | | | | | | 57.3 |
| H311W | | | | | | | | | | | W | | | | | | | | | | | | | | | | | 55.8 |
| H311F | | | | | | | | | | | F | | | | | | | | | | | | | | | | | 57.8 |
| A314M | | | | | | | | | | | M | | | | | | | | | | | | | | | | | 55.9 |
| E11P | | | | | | | | | | | | | | | | | | | | | | | P | | | | | 60.0 |
| E15P | | | | | | | | | | | | | | | | | | | | | | | | P | | | | 60.7 |
| I18P | | | | | | | | | | | | | | | | | | | | | | | | | P | | | 58.4 |
| H22F | | | | | | | | | | | | | | | | | F | | | | | | | | | | | 61.7 |
| H22Y | | | | | | | | | | | | | | | | | Y | | | | | | | | | | | 61.2 |
| H22W | | | | | | | | | | | | | | | | | W | | | | | | | | | | | 60.7 |
| A29P | | | | | | | | | | | | | | | | | | | | | | | | | | P | | 60.1 |
| H30W | | | | | | | | | | | | | | | | | | | | | | W | | | | | | 59.9 |
| S40A | | | | | | | | | | | | | | | | | | A | | | | | | | | | | 58.9 |
| Q64F | | | | | | | | | | | | F | | | | | | | | | | | | | | | | 61.6 |
| Q64W | | | | | | | | | | | | W | | | | | | | | | | | | | | | | 62.1 |
| Q64Y | | | | | | | | | | | | Y | | | | | | | | | | | | | | | | 62.3 |
| Q64P | | | | | | | | | | | | P | | | | | | | | | | | | | | | | 58.6 |
| S67A | | | | | | | | A | | | | | | | | | | | | | | | | | | | | 59.6 |
| S67T | | | | | | | | T | | | | | | | | | | | | | | | | | | | | 60.2 |
| G68L | | | | | | | | | L | | | | | | | | | | | | | | | | | | | 63.6 |
| G68Y | | | | | | | | | Y | | | | | | | | | | | | | | | | | | | 62.3 |
| D71P | | | | | | | | | | P | | | | | | | | | | | | | | | | | | 63.4 |
| T91I | | | | | | | | | | | | | I | | | | | | | | | | | | | | | 61.5 |
| T91V | | | | | | | | | | | | | V | | | | | | | | | | | | | | | 59.3 |
| S93A | | | | | | | | | | | | | | A | | | | | | | | | | | | | | 59.5 |
| S110W | | | | | | | | | | | | | | | | | | | W | | | | | | | | | 59.6 |
| S110R | | | | | | | | | | | | | | | | | | | R | | | | | | | | | 62.9 |
| E111L | | | | | | | | | | | | | | | | | | | | L | | | | | | | | 58.1 |
| E111Q | | | | | | | | | | | | | | | | | | | | Q | | | | | | | | 58.7 |
| E113Q | | | | | | | | | | | | | | | Q | | | | | | | | | | | | | 62.5 |
| E113N | | | | | | | | | | | | | | | N | | | | | | | | | | | | | 62.3 |
| E113W | | | | | | | | | | | | | | | W | | | | | | | | | | | | | 63.9 |
| E113F | | | | | | | | | | | | | | | F | | | | | | | | | | | | | 63.4 |
| E113L | | | | | | | | | | | | | | | L | | | | | | | | | | | | | 63.5 |
| H114F | | | | | | | | | | | | | | | | | | | | | F | | | | | | | 61.2 |
| H114W | | | | | | | | | | | | | | | | | | | | | W | | | | | | | 45.6 |
| H114Y | | | | | | | | | | | | | | | | | | | | | Y | | | | | | | 61.0 |
| H114I | | | | | | | | | | | | | | | | | | | | | I | | | | | | | 60.4 |
| H114N | | | | | | | | | | | | | | | | | | | | | N | | | | | | | 59.3 |
| H114Q | | | | | | | | | | | | | | | | | | | | | Q | | | | | | | 60.2 |
| L116W | | | | | | | | | | | | | | | | W | | | | | | | | | | | | 62.7 |
| L116R | | | | | | | | | | | | | | | | R | | | | | | | | | | | | 60.5 |

**Table S2. Rare HA amino acids and frequencies compared to consensus before and after repair**

| **Strain** | **Repair mutations** | **Old frequency** (%) | **New frequency** (%) |
|---|---|---|---|
| B/Ohio/01/2005 | HA1 E80R | 0.3 | 56.9 |
| | HA1 A196T | 1.0 | 91.9 |
| B/Florida/04/2006 | HA1 K88R | 0.5 | 99.2 |
| | HA1 S227G | 0.8 | 62.9 |

**Table S3. Characterization of additional HA substitutions in the stem-switch and repulsive cluster region**

| Construct | Position | | | | Results | |
|---|---|---|---|---|---|---|
| | 40 | 110 | 113 | 114 | Trimer Peak Area (mAU*mL) | Tm50 (°C) |
| --W- (Benchmark) | - | - | W | - | 6.4 | 72.0 |
| AK-W | A | K | - | F | 6.1 | 70.9 |
| AL-F | A | L | - | F | 7.0 | 70.3 |
| AR-F | A | R | - | F | 7.1 | 72.2 |
| AW-F | A | W | - | F | 8.4 | 70.3 |
| -W-F | - | W | - | F | 8.3 | 69.3 |
| AKWF | A | K | W | F | 5.4 | 73.8 |
| ALWF | A | L | W | F | 6.2 | 73.9 |
| ARWF | A | R | W | F | 7.5 | 74.9 |
| AWWF | A | W | W | F | 7.8 | 73.2 |
| -WWF | - | W | W | F | 6.5 | 72.4 |
| AKQF | A | K | Q | F | 4.9 | 71.8 |
| ALQF | A | L | Q | F | 7.4 | 71.7 |
| ARQF | A | R | Q | F | 7.5 | 73.2 |
| AWQF | A | W | Q | F | 8.1 | 71.5 |
| -WQF | - | W | Q | F | 8.0 | 70.6 |

**Table S4. Data collection, reconstruction, and model refinement statistics**

| | Influenza B virus HA at neutral pH EMD-42060, PDB: 8UAD | Influenza B virus HA at low pH EMD-43273* |
|---|---|---|
| Data collection | | |
| Microscope | Glacios | Glacios |
| Voltage (keV) | 200 | 200 |
| Nominal magnification | 150,000 x | 150,000 x |
| Exposure navigation | Image Shift | Image Shift |
| Electron exposure (e /Å$^2$) | 40.0 | 40.0 |
| Dose rate (e/pixel/sec) | 5.3 | 5.3 |
| Detector | Falcon III | Falcon IV |
| Pixel size (Å)* | 0.948 | 0.91 |
| Defocus range (µm) | -0.8 to -2.4 | -0.8 to -2.4 |
| Micrographs Used | 5,974 | 28,334 |
| Final Refined particles (no.) | 481,814 | 1,185,383 |
| | | |
| Reconstruction | | |
| Symmetry imposed | C3 (NU Refinement) | C3 (NU Refinement) |
| Resolution (global) | | |
| FSC 0.143 | 2.72 Å | 2.59 Å |
| Applied B-factor (Å$^2$) | -116.5 | -101.2 |
| | | |
| Refinement | | |
| R.m.s deviations | | |
| Bond lengths (Å) | 0.0085 | |
| Bond angles (º) | 0.93 | |
| Ramachandran | | |
| Outliers | 0.00 % | |
| Allowed | 3.55 % | |
| Favored | 96.45 % | |
| Poor rotamers (%) | 0.71 % | |
| MolProbity score | 1.44 | |
| EMRinger score | 3.97 | |
| Clashscore (all atoms) | 4.28 | |

***** only reconstructed EM density maps deposited due to insufficient density around the stem part at low pH.

## **Supplementary datafile:** CMP scores

The file contains Rosetta energy score outputs generated using the Coupled Move Protocol (CMP) for all possible substitutions at every position in the influenza B HA protein, based on PDB structures 4m44, 4nrj, and 6fyw.

**Supplementary Methods**

Intact mass LC-MS

Samples containing influenza B virus HA proteins were prepared for Liquid Chromatography-Mass Spectrometry (LC-MS) analysis by reducing disulfide bridges with or without deglycosylation procedure. Reduction of disulfides was done by adding Tris(2-carboxyethyl)phosphine) (TCEP) (Thermo Scientific) to a final concentration of 50 mM and incubating at 70°C for 30 min while shaking. To remove N-linked glycans, 50 µg of protein was incubated at 75°C for 15 min, followed by addition of 2 µL of Rapid PNGase F (Promega) and incubation for 1 hour at 50°C. Prior to analyses, all samples were diluted with 0.1% formic acid to the final concentration of 0.1 g/L, and 0.2 µg of protein was injected for separation and mass detection.

All intact mass LC-MS measurements were performed on the Waters H-class ultra high-performance liquid chromatography (UHPLC) equipped with FLR and PDA detectors and coupled to Waters Xevo G2-S quadrupole Time-of-Flight (Q-ToF) mass spectrometer. Data acquisition was done by using MassLynx V4.2 software. Proteins were desalted and separated on Acquity UHPLC Protein BEH C4 guard column in tandem with Acquity UHPLC Protein BEH C4 Column (300A, 1.7µm, 1 mm x 150 mm), both kept at 80°C. Elution was achieved with 14 min gradient (20-60% mobile phase B, flow rate 0.1 mL/min). 0.1% formic acid (FA) in Milli-Q water (LC-MS grade) and 0.1% FA in acetonitrile (ACN) were used as mobile phases A and B, respectively. Fluorescence was measured by monitoring emission at 340 nm following excitation at 280 nm. UV absorbance was measured at 220 and 280 nm. Xevo G2-S Q-ToF mass spectrometer was operated in positive ion mode and in sensitivity mode. Acquisition parameters were set as follows: mass range – 500-2500 m/z, capillary – 2.5 kV, sampling cone – 40 V, source temperature – 100°C, source offset – 80 V, desolvation temperature – 350°C, cone gas flow – 0 L/hr, desolvation gas flow – 700 L/hr. Mass calibration was performed using NaI in the range 100-2500 m/z, and Leu-enkephalin was introduced via lockmass channel for mass correction. Deconvolution of charge state envelopes corresponding to HA proteins was done in UniDec V5.0.5 (https://github.com/michaelmarty/UniDec) by combining spectra over the entire chromatographic peak for HA1 and HA2 components. For deglycosylated HA1, default parameters were used except for the following: m/z range – 500-2000, background subtraction – 100, charge range – 10-100, mass range – 35000-60000 Da, sample mass every (Da) – 0.1, smooth nearby points – none, suppress artifacts – none, peak FWHM (Th) – 1.0, picking range (Da) – 50, picking threshold – 0.1. For glycosylated HA1, following settings facilitated deconvolution of highly congested mass spectra: smooth nearby points – lots, suppress artifacts – lots. For HA2, parameters were adjusted accordingly: charge range – 5-60, mass range – 19000-24000 Da, smooth nearby points – some, suppress artifacts – none. All additional data processing and visualizations were made in R supplemented with ggplot2 package.
